# Construction of a fine-grained retrieval model for archival text-image based on the integration of scene graph generation and attention mechanism

**DOI:** 10.1371/journal.pone.0353505

**Published:** 2026-07-24

**Authors:** Mengyuan Zhang

**Affiliations:** School of Continuing Education, Sichuan Vocational and Technical College, Suining, China; National Textile University, PAKISTAN

## Abstract

To tackle the challenges in fine-grained retrieval stemming from noise, official seal occlusions, small text blocks, and other issues prevalent in archival text images, and to fulfill the requirements of integrating both textual and visual dual features while enhancing retrieval accuracy and efficiency, this study has devised a five-tier architectural model. This model comprises an input layer, a preprocessing layer, a scene graph generation layer, an attention fusion layer, and a retrieval matching layer. The model incorporates a dedicated scene graph generation module tailored for archival data, aiming to enhance element detection. Additionally, it features a three-tier attention fusion module that integrates scene graph, text, and cross-modal features to ensure precise feature alignment. Training is carried out using a multi-task loss function, and an index is created to streamline retrieval and matching processes. Experimental results show that the proposed model achieves a Top-1 accuracy of 83.7% and an average precision of 88.3% on the test set, representing a 25.1% improvement over the Top-1 accuracy of an optical character recognition (OCR) combined with word frequency and inverse document frequency model. The proposed model achieves a Top-1 accuracy of 6.1% higher than the archival retrieval network model for examples with official seal occlusion and a Top-1 accuracy of 76.8% for small text blocks. The response time for a single retrieval is 52.6ms. Research provides technical support for efficient retrieval of large-scale archives in archives, effectively solving the problem of archive retrieval in complex scenarios, and significantly improving the efficiency of archive management and utilization.

## 1. Introduction

With the continuous advancement of the digital transformation of archive management, the stock of archival text images has exploded, and the demand for fine-grained retrieval has become increasingly prominent [1]. Compared with traditional coarse-grained retrieval, which only focuses on matching the overall content, fine-grained retrieval emphasizes the accurate capture and distinction of local key information in the image. It also requires in-depth exploration of the subtle differences in text content, visual elements, and their relationships at the semantic level. For archival text image scenes, fine-grained retrieval needs to focus on identifying detailed features such as official seal form, small text block semantics, layout differences, and annotation traces, to meet the core demands of detail and accuracy in scenes such as accurate archival research and voucher verification [2]. At present, research on archival text image retrieval has made certain progress. Traditional methods mostly rely on manually annotated text features or shallow visual features, which are difficult to effectively capture complex semantic associations [3]. The scene graph generation technology that has emerged in recent years provides a new path for semantic-level retrieval by constructing structured semantic representations of entities and relationships. Meanwhile, the attention mechanism can dynamically focus on key visual areas and enhance feature differentiation [4]. However, the integrated application of the two in the archival field still has obvious shortcomings. Scene graphs generate multifaceted, general images, but their ability to model specific knowledge about archive categories, signature semantics, and textual logical relationships is limited. Attention mechanisms typically focus solely on visual features and don’t fully capture the deep semantic connections within scene graphs. As a result, when trying to distinguish subtle differences between similar types of archives, the retrieval accuracy and efficiency fall short of meeting real-world application needs [5,6]. Against this backdrop, constructing a fine-grained retrieval model for archival document images that integrates scene graph generation and the attention mechanism has become essential. This approach serves as a critical exploration direction to address current industry challenges and enhance the intelligence level of archival retrieval.

C. M. Lo et al. extracted high-level features by fine-tuning the deep learning architecture for medical image retrieval, and used three different annotated images for classification and query. The results showed that the classification accuracy of this method reached 94% [7]. However, the above-mentioned methods are highly dependent on domain-specific labeled data and do not take into account the semantic alignment issue between text and images, making it difficult to be transferred to multimodal and strongly structured scenarios such as archival text and images. G. Shamsipour et al. proposed combining the manual features with the output of the residual block of the deep neural network in the feature fusion stage for manual feature image retrieval, removing the layers performing the classification task in the deep network and transforming the feature vector. The results showed that the accuracy of this method on the Corel-1k and Corel-5k datasets was 96.68% and 94.56% respectively [8]. However, this method relies on manually designed visual features and lacks robustness against common degradation problems such as noise, occlusion, and small text blocks in archival images. M. H. Hadid et al. proposed an image retrieval model combined with singular value decomposition technology for content-based image retrieval. The model first extracted features of the query image and the database image, then used singular value decomposition to reduce the dimensionality, and finally compared the similarity using the cosine metric. The results showed that the average accuracy of the model on the Corel-1K dataset was 0.948 [9]. Although this method has high computational efficiency, its feature representation ability is limited and it cannot capture the rich semantic relations and layout structures in archival images. The above-mentioned methods generally focus on feature extraction or shallow fusion of a single modal, failing to effectively integrate text and visual information, nor do they take into account the structured semantic representation within the image. Archival images have a strong text-visual symbiosis characteristic. Relying solely on visual features or simple text recognition, such as optical character recognition (OCR), is prone to retrieval failure due to issues such as noise and occlusion. L. Du et al. proposed a method for contextual analysis and retrieval of historical images in digital visual archives. They used computer vision to crop historical images from picture magazines, trained a machine learning model on the cropped images and the original historical photo dataset, and compared the image similarities using a collection of visual transformers. The results showed that the model achieved a 77.8% retrieval accuracy for the top 15 images in the evaluation dataset [10]. T. Yao et al. proposed a simple yet effective image-text retrieval method, called cross-modal interactive reasoning, for enhancing visual language pre-training. Extensive experiments showed that the research method achieved 52 and 97.5 results on two widely-used datasets compared to the state-of-the-art non-pre-trained methods, and it also outperformed several mainstream fine-tuned visual language pre-trained models [11]. Although cross-modal pre-trained models perform well in general retrieval tasks, they have the problem of semantic confusion in archival image retrieval, that is, the models have difficulty understanding the specific semantic logic of archives, such as the affiliation relationship between document numbers and main text, and the positional relationship between seals and titles. In addition, most historical image retrieval methods focus on visual content and do not fully integrate structured text information. N. Felemban et al. designed an effective method and system, called EDIR, which can answer these queries while taking into account the bandwidth limitations encountered in wireless networks and the limited energy and computing power on mobile devices. The results showed that, compared with other methods, EDIR reduced the amount of transmitted data by more than 45% while achieving a good F1 value [12]. Z. Zhuo et al. explored the semi-supervised domain adaptive remote sensing image retrieval method and constructed a CNN based on gabor, enabling the network to effectively capture the texture information of images. Secondly, a cross-domain knowledge transfer strategy based on dual Gabor neural network learning is proposed. Thirdly, an unsupervised random feature mapping method based on probabilistic distance is proposed. The results show that this method significantly improves the retrieval accuracy in the target domain and achieves better retrieval accuracy [13]. Most of the existing efficient retrieval methods focus on optimization at the system level, while they are still relatively weak at the semantic representation level. The domain adaptation and texture enhancement techniques used in remote sensing image retrieval can provide inspiration for element detection in archival images, but cross-modal alignment still needs to be combined with text semantics.

Therefore, the study proposes a fine-grained retrieval model for archival text images that integrates scene graph generation and attention mechanisms. This model adopts a five-layer architecture, including the input layer, preprocessing layer, scene graph generation layer, attention fusion layer and retrieval matching layer. It optimizes element detection by constructing a dedicated scene graph module for archives and designs a three-level attention mechanism to achieve deep integration of visual and text features. The innovation of this research primarily manifests in its tailored solution to the fine-grained retrieval requirements of archival text images, accomplished through the development of a multi-stage architecture that seamlessly integrates textual and visual features. This architecture utilizes an archive-specific scene graph generation module to accurately extract key elements and their interrelationships, while a multi-dimensional attention mechanism is implemented to facilitate a profound fusion of cross-modal features. Ultimately, retrieval accuracy and efficiency are notably enhanced through a retrieval matching module that combines feature similarity and structural matching. The key contributions of this research are as follows: To address challenges such as small text blocks and seal occlusions in archival images, customized node definitions and prior box optimization strategies have been devised to improve the detection accuracy of key elements. These strategies are considered effective in enhancing the precision of element identification within complex archival contexts. Furthermore, by integrating the Graph Attention Network (GAT) with the BERT encoder, semantic alignment between scene graph features and text features has been achieved, thereby enriching the cross-modal feature representation. This integration is viewed as a step forward in bridging the gap between visual and textual information in archival retrieval. Additionally, a comprehensive ranking approach has been adopted by combining feature similarity and structural similarity. This approach is believed to ensure retrieval accuracy while also improving response times, offering a balanced solution to the demands of efficient and accurate archival retrieval. The practical implications of this research are significant, as it contributes to enhancing the efficiency and intelligence level of archive management. It supports a variety of archival application scenarios, including historical archive research, digital preservation, and cross-modal archive searches. Moreover, it is anticipated to facilitate the digital transformation of archives and the upgrading of knowledge services, providing a valuable reference for future advancements in the field. The research structure mainly includes five sections: Section 1 systematically elaborates on the research background, practical needs, and limitations of existing methods for fine-grained retrieval of archival text images, clarifying research objectives and innovative directions. The second section constructed a five stage retrieval model architecture. The third section is the validation of the effectiveness and feasibility of the research method. Section 4 provides an in-depth analysis of the effectiveness of the model mechanism, compares the advantages and limitations of existing methods, and proposes future directions for scalability. Section 5 summarizes the research results and practical application value.

## 2. Research methods

### 2.1. Structure of the fine-grained retrieval model for archive text-image

**Fig 1:**
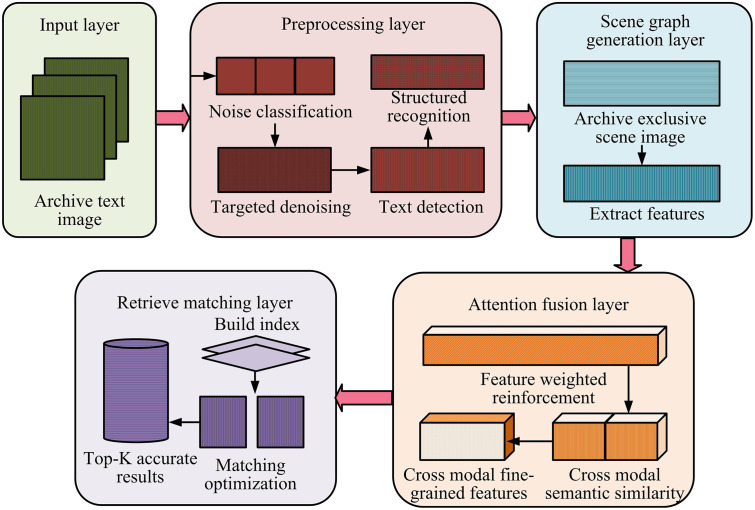
The architecture of fine-grained retrieval model for archive text-images.

To address the demand for the fusion of visual and text features in archival text images, this subsection designs a five-stage retrieval model architecture. The architecture includes an input layer, a preprocessing layer, a scene image generation layer, an attention fusion layer, and a retrieval matching layer. It aims to achieve the collaborative functions of each layer to realize fine-grained feature extraction and matching, spanning from images to semantics and from single-modal to cross-modal. The overall architecture of the archive text-image fine-grained retrieval model is shown in Fig 1.

In Fig 1, the input layer receives single-modality archive text-images, supports common formats, and records the basic attributes of the images. The preprocessing layer adopts a four step process, including noise classification, targeted denoising, text detection, and structured recognition. Firstly, it distinguishes between salt and pepper noise and Gaussian noise through a noise statistical model, and uses median filtering and Gaussian filtering respectively for denoising. The noise statistical model is based on the local grayscale statistical characteristics of the image: salt and pepper noise is manifested as randomly appearing extremely bright or dark pixels, with grayscale values close to 0 or 255, and significant differences in grayscale from neighboring pixels; Gaussian noise is manifested as random fluctuations in the normal distribution of grayscale values, with large local variances but relatively continuous grayscale value ranges. When making specific judgments, calculate the grayscale range and variance within the local window (3 × 3 pixels) of the image. If the range is large and there are extreme grayscale values (<5 or>250), it is judged as salt and pepper noise; If the variance is large but there are no extreme grayscale values, it is judged as Gaussian noise. Subsequently, EAST is used to locate the text block area. Finally, text recognition is achieved through optimized Tesseract OCR, which outputs clean images and structured text, including three types of structured information: title, body, and number. The scene graph generation layer takes the clean images as input, constructs archive-specific scene graphs, and extracts visual and textual features of nodes as well as relational semantic features of edges, providing a visual-semantic foundation for cross-modal fusion. The attention fusion layer employs a three-level attention mechanism to weight and enhance the scene graph features and textual features respectively. It then calculates cross-modal semantic similarity to achieve precise alignment between key visual semantics and core textual semantics, outputting cross-modal fine-grained features. The retrieval matching layer stores the fused features in a vector database to construct an index. Through similarity calculation and structural matching optimization, it achieves efficient matching between queries and archive images, returning Top-K accurate results [14].

### 2.2. Design of the archive-specific scene graph generation module

In response to special issues such as small text blocks and seal occlusion in archival images, this small section has designed a scene graph generation module for archives, which includes three parts: node definition and detection optimization, relationship definition and prediction, and graph structure optimization. The research categorizes archive elements into four types of core nodes. Text block nodes include subcategories such as titles, main texts, and serial numbers, with features defined as a combination of bounding box coordinates, word embeddings of the recognized text, and grayscale mean values [15]. Seal nodes are characterized by circular bounding box radii, a red channel proportion exceeding 60%, and positions predominantly located in the upper right corner of the image. Table nodes are defined by the number of horizontal and vertical grid lines detected, the number of cells, and bounding box coordinates. Blank area nodes are identified by a continuous region devoid of text or seals with an area exceeding 500px², coupled with a grayscale variance below 10 [16,17]. To address the insufficient detection accuracy of small text blocks and seals in archives, the research incorporates archive element prior boxes into the Region Proposal Network (RPN) of the Faster Region-based Convolutional Neural Network (Faster R-CNN). The scales and aspect ratios of these prior boxes are designed based on the statistical characteristics of archive elements, with the scale calculation shown in Equation (1) [18].


$$\begin{array}{c} s_{k}=s_{min}+(s_{max}-s_{min})\cdot \frac{k-1}{m-1} \end{array}$$ (1)

**Fig 2:**
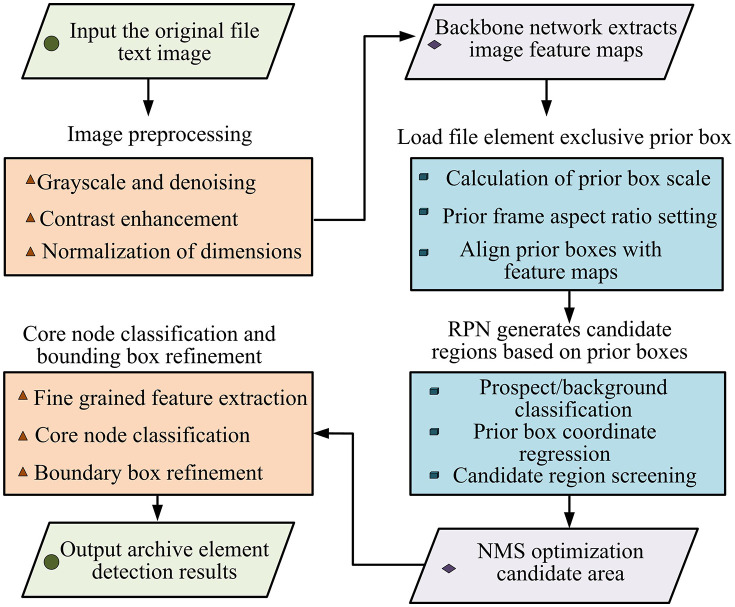
The improved detection process.

In Equation (1), $s_{k}$ is the scale (pixel) of the k th prior, representing the ratio of the edge length of the prior box to the edge length of the image feature map; $s_{min}$ is the minimum prior box scale; $s_{max}$ is the maximum prior box scale; k is the prior box index; m is the number of prior boxes. The aspect ratios of the prior boxes are designed into three categories: 1:1 (suitable for seals and small text blocks), 2:1 (suitable for main text blocks), and 1:2 (suitable for table rows), ensuring coverage of the morphological characteristics of archive elements [19]. The improved detection process is illustrated in Fig 2. By constraining the generation of candidate regions in the RPN with prior boxes, the number of candidate boxes for non-archive elements is reduced, and the detection accuracy for small elements is enhanced.

The research defines four core types of relationships by integrating archive layout and semantic logic. The containment relationship indicates that one element contains another, such as a title containing keywords or a table containing text blocks. The positional relationship refers to the relative positions of elements within the layout, for example, a seal being located in the upper right corner of a title and a serial number being positioned on the left side of the main text. The associative relationship denotes the semantic connections between elements, such as the semantic association between the main text and a table, or a serial number and a title belonging to the same archive item. The subordination relationship signifies hierarchical or subordinate connections between elements, such as a sub-table being subordinate to a main table or an attachment number being subordinate to the main archive number [20,21]. These four types of relationships collectively form the basic logical framework among archive elements. The research employs a node feature concatenation method and a GAT architecture to predict relationships between nodes. Initially, the visual and textual features of any two nodes are concatenated, as shown in Equation (2).


$$\begin{array}{c} h_{ij}\text{=}h_{i}^{v}⊕h_{j}^{v}⊕h_{i}^{t}⊕h_{j}^{t} \end{array}$$ (2)

In Equation (2), $h_{ij}$ is the concatenated feature vector of nodes $V_{i}$ and $V_{j}$; $h_{i}^{v}$ and $h_{j}^{v}$ are the visual feature vectors of nodes $V_{i}$ and $V_{j}$, respectively; $h_{i}^{t}$ and $h_{j}^{t}$ are the text feature vectors of nodes $V_{i}$ and $V_{j}$, respectively; ⊕ represents vector concatenation operation. The main reason for using node feature concatenation is that the relationship types in the archive scene graph are relatively fixed, and the semantic associations between nodes have strong regularity. In such highly structured scenarios, simple feature concatenation combined with learnable attention weight vectors can effectively capture the strength of relationships between nodes. Introducing more complex relational reasoning modules (such as graph neural networks+logical reasoning layers, neural symbol systems, etc.) may enhance the richness of relational expression, but it will significantly increase computational complexity, which is not conducive to the efficient retrieval needs of million level archives. The attention mechanism of GAT is utilized to learn the importance of associations between nodes, with the weight calculation shown in Equation (3).


$$\begin{array}{c} \alpha_{ij}={\text{softmax}}_{j}\left(LeakyReLU\left(a^{T}\cdot h_{ij}\right)\right) \end{array}$$ (3)

In Equation (3), $\alpha_{ij}$ represents the attention weight of nodes $V_{i}$ and $V_{j}$; a is a learnable attention weight vector; LeakyReLU is the activation function, with a negative slope value of 0.2; ${\text{softmax}}_{j}$ represents normalizing all nodes j adjacent to $V_{i}$. In relationship classification, multiplying $h_{ij}$ with $\alpha_{ij}$ and inputting it into the fully connected layer outputs the probabilities of four types of relationships, as shown in Equation (4).


$$\begin{array}{c} p_{ij}^{r}=\text{softmax}(W_{r}\cdot (\alpha_{ij}\cdot h_{ij})+b_{r}) \end{array}$$ (4)

**Fig 3:**
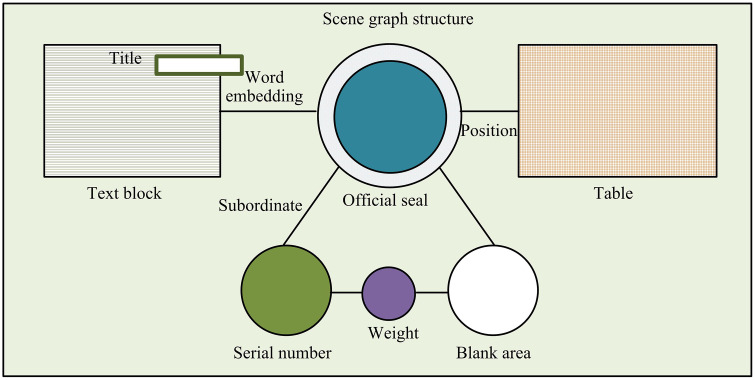
Optimized scene graph structure.

In Equation (4), $p_{ij}^{r}$ is the probability that nodes $V_{i}$ and $V_{j}$ belong to the r th type of relationship; $W_{r}$ is the weight matrix for relationship classification; $b_{r}$ is the bias vector; softmax is the normalization function. To reduce redundant information, the research optimizes the graph structure through node attention weighting and redundant edge pruning. During the node attention weighting process, higher weights are assigned to key nodes such as serial number nodes and seal nodes, with a weight coefficient of 0.8; moderate weights are given to title nodes and table nodes, with a coefficient of 0.5; and lower weights are applied to main text nodes and blank area nodes, with a coefficient of 0.2. These weight coefficients are used to enhance the semantic representation of key nodes during subsequent feature fusion [22]. Redundant edges include edges connecting blank areas and edges with relationship strength below a threshold and non core relationships, which are pruned [23]. The optimized scene graph structure is shown in Fig 3.

### 2.3. Design of multi-dimensional attention fusion module

**Fig 4:**
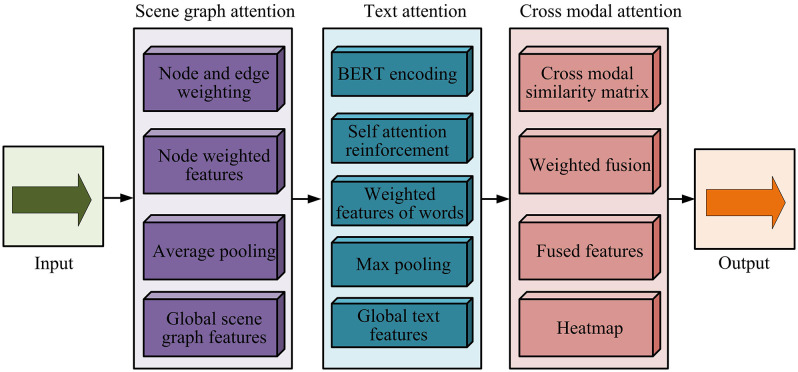
Attention module structure.

To deeply integrate scene graph and text features, this subsection proposes a three-level attention fusion mechanism comprising scene graph attention, text attention, and cross-modal attention, as shown in Fig 4. Scene graph attention uses GAT to weight key nodes and edges, extracting global scene graph features. Textual attention employs Bidirectional Encoder Representations from Transformers (BERT) with a self-attention mechanism to emphasize keywords, generating global textual features. Cross-modal attention computes a similarity matrix to fuse the two features, outputting fine-grained fused features.

Based on GAT, nodes and edges in the scene graph are weighted to highlight key nodes such as serial number nodes, seal nodes, and table nodes, as well as core relationships like containment and subordination. The weighted node features are represented as shown in Equation (5).


$$\begin{array}{c} v_{i}^{g}=\sum_{j\in N\left(i\right)}\alpha_{ij}\cdot (w_{i}\cdot h_{i}^{v}+w_{j}\cdot h_{j}^{v}) \end{array}$$ (5)

In Equation (5), $v_{i}^{g}$ represents the weighted scene graph feature vector of node $V_{i}$; N(i) denotes the set of nodes directly connected to node $V_{i}$; and $h_{i}^{v}$ and $h_{j}^{v}$ are the visual feature vectors of the nodes, respectively. The number of nodes contained in different archive images varies greatly. For example, a simple archive may only contain 2–3 nodes, while a complex archive may contain more than 10 nodes, and the contribution of each node to the retrieval task is different. To convert these variable length node level feature sequences into fixed dimensional global scene graph features, pooling operations are required for aggregation. The study chose average pooling as the aggregation method, which focuses on calculating the mean of the weighted feature vectors of all nodes in the node dimension. This strategy can uniformly integrate the information of all nodes, avoiding the dominance of global representation due to significant local features of certain nodes, thereby preserving the overall structural semantics. In contrast, max pooling only retains the maximum values of node features in each dimension. Although it can highlight the most significant local responses, it may lose the distribution information of other nodes, especially when there are multiple equally important key nodes in the archive image. Max pooling may lead to information bias. In addition, average pooling is less sensitive to fluctuations in node features and can more stably reflect the overall structure of the scene graph in situations where the number of nodes varies greatly. The study employs the BERT model to encode structured text, enhancing the features of keywords such as archive serial numbers and retention periods through a self-attention mechanism. The calculation of self-attention weights is shown in Equation (6) [24].


$$\begin{array}{c} \alpha_{t}^{mn}={\text{softmax}}_{n}\left(\frac{Q_{m}\cdot \kappa_{n}^{T}}{\sqrt{d_{k}}}\right) \end{array}$$ (6)

In Equation (6), $\alpha_{t}^{mn}$ is the self-attention weight of the m th word in the text; $Q_{m}$ is the query vector of the m th word; $\kappa_{n}$ is the key vector of the n th word; $d_{k}$ is the dimension of the query/key vector, which is used to alleviate the gradient disappearance caused by excessive inner product; ${\text{softmax}}_{n}$ represents normalization of all n. The weighted text features are shown in Equation (7) [25].


$$\begin{array}{c} v_{m}^{t}=\sum_{n=1}^{L}\alpha_{t}^{mn}\cdot V_{n}^{t} \end{array}$$ (7)

In Equation (7), $v_{m}^{t}$ is the weighted text feature vector of the m th word; L is the number of words in the text; $V_{n}^{t}$ is the original BERT encoding vector of the n th word. Maximum pooling is performed on all $v_{m}^{t}$ to obtain the global text feature $V_{t}$. For the attention matrix of weighted scene graph features and weighted text features, the semantic similarity between the two is calculated to achieve cross-modal feature alignment. First, the similarity matrix is calculated as shown in Equation (8).


$$\begin{array}{c} M=\frac{V_{g}\cdot V_{t}^{T}}{\sqrt{d}} \end{array}$$ (8)

In Equation (8), M is the cross-modal attention matrix; $V_{g}$ is the global scene graph feature; $V_{t}$ is the global text feature; d is the mean value of the feature dimension, which is used to normalize the similarity value. The study takes the weight λ corresponding to the maximum value in M, performs weighted fusion of scene graph features and text features, and obtains cross-modal fine-grained fusion features, as shown in Equation (9).


$$\begin{array}{c} V_{f}=\lambda \cdot V_{g}+(1-\lambda )\cdot V_{t} \end{array}$$ (9)

In Equation (9), $V_{f}$ is the cross-modal fine-grained fusion feature; λ is the cross-modal attention weight.

### 2.4. Search matching module design

**Fig 5:**
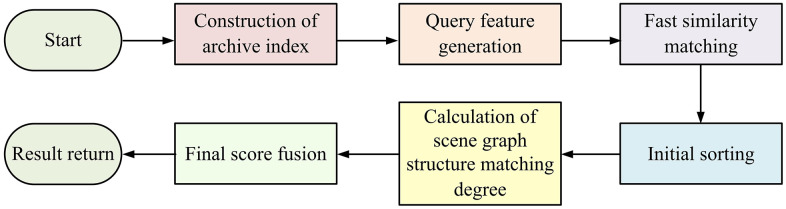
Search matching module process.

To enhance the efficiency and accuracy of retrieval, this section designs a retrieval matching module based on feature similarity and scene graph structure matching, which supports rapid indexing and sorting of large-scale archives. The search and matching module process is shown in Fig 5.

In Fig 5, the study stores the cross-modal fusion features of all images in the archive into the Facebook AI Similarity Search (FAISS) vector database, using the Inverted File with Flat Storage (IVF_FLAT) index type (suitable for million-level archives). First, the features are clustered into 100 cluster centers. When querying, only the 10 cluster centers closest to the query features are searched to improve the retrieval speed. When building the index, the scene graph structure information (node type, relationship type) of each archive is also stored. The corresponding cross-modal fusion feature $V_{q}$ is generated according to the query type (text query/image query), and the cosine similarity is used to calculate the matching degree between $V_{q}$ and the archive feature $V_{f,a}$, as shown in Equation (10).


$$\begin{array}{c} S_{cos}=\frac{V_{q}\cdot V_{f,a}^{T}}{\|V_{q}\|\cdot \|V_{f,a}\|} \end{array}$$ (10)

In Equation (10), $S_{cos}$ is the cosine similarity; $V_{q}$ is the cross-modal fusion feature of the query; $V_{f,a}$ is the cross-modal fusion feature of the a th archive; and ‖·‖ is the L2 norm (the modulus of the vector). In the result sorting and optimization phase, a comprehensive evaluation is performed by combining feature similarity and scene graph structural matching. First, the initial sorting results are obtained by sorting in descending order based on $S_{cos}$. The structural graph matching between the query scene graph $G_{q}=\left(V_{q},E_{q}\right)$ and the archive scene graph $G_{a}=\left(V_{a},E_{a}\right)$ is calculated as shown in Equation (11).


$$\begin{array}{c} S_{\text{stru}}=\frac{\left|V_{q}⋂V_{a}\right|}{\left|V_{q}⋃V_{a}\right|}\cdot \frac{\left|E_{q}⋂E_{a}\right|}{\left|E_{q}⋃E_{a}\right|} \end{array}$$ (11)

In Equation (11), $S_{\text{stru}}$ is the structural similarity; $\left|V_{q}⋂V_{a}\right|$ is the number of common nodes between the query and the archive; $\left|V_{q}⋃V_{a}\right|$ is the total number of nodes between the query and the archive; $\left|E_{q}⋂E_{a}\right|$ is the number of common relationships; and $\left|E_{q}⋃E_{a}\right|$ is the total number of relationships. In the final ranking, $S_{cos}$ and $S_{\text{stru}}$ are weighted and fused, as shown in Equation (12).


$$\begin{array}{c} S_{\text{final}}=0.7S_{cos}+0.3S_{\text{stru}} \end{array}$$ (12)

In Equation (12), $S_{\text{final}}$ is the final score. Finally, the top-K results are returned in descending order according to $S_{\text{final}}$. Among them, the feature similarity weight is set to 0.7, and the structure similarity weight is set to 0.3. This weight combination is obtained through grid search optimization on the validation set. This weight setting reflects the dominant role of feature similarity in retrieval and matching. Structural similarity serves as a supplementary constraint, effectively enhancing the ability to distinguish archival images with similar visual features but significant structural differences.

### 2.5. Model training strategy

To ensure that all modules of the model work together, we designed a training process that combines multi-task loss functions, step-by-step pre-training, and end-to-end fine-tuning to avoid gradient explosion in the early stages of training and improve model convergence stability. We used a multi-task loss consisting of scene graph generation loss, cross-modal fusion loss, and retrieval matching loss. The total loss is calculated as shown in Equation (13).


$$\begin{array}{c} L_{\text{total}}=\lambda_{\text{1}}L_{\text{sg}}+\lambda_{\text{2}}L_{\text{cm}}+\lambda_{\text{3}}L_{\text{ret}} \end{array}$$ (13)

In Equation (13), $L_{\text{total}}$ is the total loss, $\lambda_{\text{1}}$, $\lambda_{\text{2}}$, and $\lambda_{\text{3}}$ are loss weights; $L_{\text{sg}}$ is the scene graph generation loss; $L_{\text{cm}}$ is the cross-modal fusion loss; and $L_{\text{ret}}$ is the retrieval matching loss. The scene graph generation loss includes node classification loss and relationship prediction loss, both of which use cross-entropy loss [26]. The node classification loss $L_{\text{node}}$ is calculated as shown in Equation (14).


$$\begin{array}{c} L_{\text{node}}=-\frac{\text{1}}{N}\sum_{i=1}^{N}\sum_{c=1}^{\text{4}}y_{i,c}log\left(p_{i,c}\right) \end{array}$$ (14)

In Equation (14), N is the total number of nodes; c is the node category; $y_{i,c}$ is the category label of node $y_{i,c}$; and $p_{i,c}$ is the probability that node $y_{i,c}$ is predicted to be of the same category. The relationship prediction loss $L_{\text{rel}}$ is calculated as shown in Equation (15).


$$\begin{array}{c} L_{\text{rel}}=-\frac{\text{1}}{M}\sum_{j=1}^{M}\sum_{r=1}^{\text{4}}z_{i,r}log\left(p_{j,r}\right) \end{array}$$ (15)

In Equation (15), M is the total number of edges; r is the relationship category, with 1–4 corresponding to four types of relationships; $z_{i,r}$ is the relationship label of edge j; and $p_{j,r}$ is the probability that edge $y_{i,c}$ is predicted to be relationship r. The scene graph generation loss is the sum of the node classification loss $L_{\text{node}}$ and the relationship prediction loss $L_{\text{rel}}$. The cross-modal fusion loss $L_{\text{cm}}$ uses a contrastive learning loss to make the fused features of similar samples closer and the distance between dissimilar samples farther, as shown in Equation (16).


$$\begin{array}{c} L_{\text{cm}}=-\frac{\text{1}}{N_{\text{pos}}}\sum_{\left(i,j\right)\in \Omega }log\left(\frac{exp(\text{sim}(V_{f,i},V_{f,j})/\tau )}{\sum_{k\in \Lambda_{i}}exp\left(\text{sim}\left(V_{f,i},V_{f,k}\right)/\tau \right)}\right) \end{array}$$ (16)

In Equation (16), Ω is the set of similar sample pairs; $N_{\text{pos}}$ is the number of similar sample pairs; sim(·,·) is the cosine similarity; τ is the temperature parameter; $\Lambda_{i}$ is the set of dissimilar samples to sample i; $V_{f,i}$, $V_{f,j}$, and $V_{f,k}$ are the fusion features of samples i, j, and k, respectively. The retrieval matching loss $L_{\text{ret}}$ uses a triplet loss to shorten the distance between the query sample and the positive sample and increase the distance between the query sample and the negative sample, as shown in Equation (17).


$$\begin{array}{c} L_{\text{ret}}=max(0,{\|V_{f,q}-V_{f,p}\|}^{\text{2}}-{\|V_{f,q}-V_{f,n}\|}^{\text{2}}+\Upsilon ) \end{array}$$ (17)

**Fig 6:**
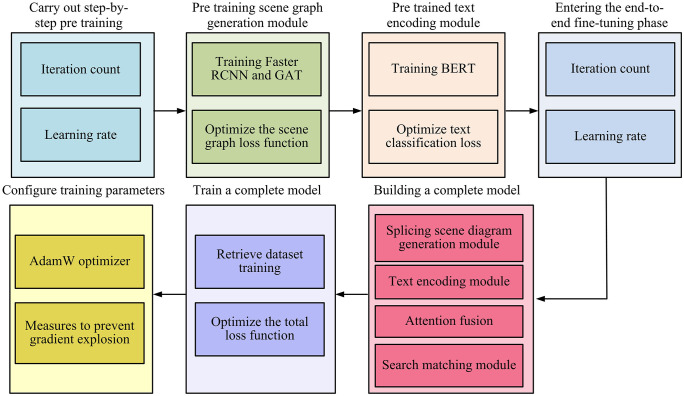
Model training process.

In Equation (17), $V_{f,q}$, $V_{f,p}$, and $V_{f,n}$ are the fused features of the query, positive sample, and negative sample, respectively; ${\|\cdot \|}^{\text{2}}$ is the square of the L2 norm; and Υ is the margin parameter (taken as 0.2 to ensure that the distance difference between positive and negative samples is greater than 0.2). The model training process is shown in Fig 6.

As shown in Fig 6, step-by-step pre-training is first performed with 100 iterations and a learning rate of 0.0001. The scene graph generation module (Faster R-CNN and GAT) is pre-trained using annotated archival images, while the text encoding module (BERT) is pre-trained on 100,000 archival texts. Subsequently, end-to-end fine-tuning is conducted with 50 iterations and a learning rate of 0.00005, combining all modules to optimize the overall loss function using the archival retrieval dataset.

## 3. Results

### 3.1. Experimental environment and dataset

The experimental hardware configuration used an Intel Xeon Gold 6348 processor with a 2.6GHz clock speed and 40 cores and 80 threads. The graphics card used two NVIDIA A100 GPUs, each with 40GB of video memory. The memory configuration was 256GB of DDR4-3200 RAM. The storage used a 2TB NVMe solid-state drive. The software environment was based on the Ubuntu 20.04 operating system, using PyTorch 1.12.0 and TensorFlow 2.9.0 as the deep learning frameworks. Text processing relied on the Hugging Face Transformers 4.24.0 library, and vector search made use of the FAISS 1.7.3 library. Data processing tools included OpenCV 4.6.0 and Pandas 1.5.3.

The experimental dataset came from the historical and modern archives of a provincial archives, containing a total of 50,000 archival text images, covering three categories: document archives (32,000 images), accounting archives (8,000 images), and scientific and technological archives (10,000 images). The image resolution ranged from 1200 × 1600 pixels to 2400 × 3200 pixels, and contained typical archival image problems such as salt and pepper noise (18%), Gaussian noise (22%), official seal occlusion (35%), and small text blocks (42%). The dataset was divided into a training set (35,000 images), a validation set (10,000 images), and a test set (5,000 images) in a ratio of 7:2:1. All images were annotated with node information (four categories: text blocks, official seals, tables, and blank areas, totaling 182,000 nodes) and relationship information (four categories: containment, location, association, and subordination, totaling 256,000 relationships). Structured text labels (title, body, number) and search similarity labels (levels 1–5, with level 1 being a complete mismatch and level 5 being a complete match) were also provided. In addition, to evaluate the generalization ability of the research model, the study introduced a city level archive in East China, which contains 15000 archival graphic and textual images, covering document archives (9000), accounting archives (3500), and scientific and technological archives (2500). The image resolution range is from 1000 × 1400 pixels to 2000 × 2800 pixels, of which 32% are samples of official seal occlusion and 38% are samples of small text blocks.

All images were annotated at multiple levels. At the node level, four core elements including text blocks, seals, tables, and blank areas were annotated, totaling 182000 nodes. On average, each image contains about 3.6 nodes, with text block nodes accounting for the highest proportion, about 62%. At the relational level, four types of semantic relationships were annotated, including relationships, orientation relationships, association relationships, and membership relationships, totaling 256000 relationships. On average, each image contained about 5.1 relationships, with orientation relationships and association relationships being the most common, accounting for 38% and 29% of the total correlation coefficients, respectively. The above annotation density ensured the integrity and semantic richness of scene graph construction. The retrieval similarity tags adopted a five level scoring system, and were independently annotated by three experts in the field of archives based on the correlation between query conditions and archive images. The final tags were based on the consensus of the majority or expert consultation results. The specific definitions of each level were as follows: Level 1 (completely unrelated) indicated that there was no correlation between the image content and the query conditions in terms of theme, elements, and structure, such as when querying the “2020 Financial Audit Report” and returning the “1985 Personnel Appointment and Removal Notice”; Level 2 (partially related) indicated that the image only had a very small number of common elements with the query criteria, but the overall theme and structure did not match, such as the query containing “seals” but returning images with only similar seals and no matching text; Level 3 (moderately correlated) indicated that the image matched the query criteria on some key elements, but there were significant differences in overall structure or semantic associations, such as matching table formats but not matching document numbers; Level 4 (highly correlated) indicated a high degree of match between the image and the query criteria in terms of primary elements and structure, with only minor differences in details such as title matching but slight offset in seal position; Level 5 (completely related) indicated that the image and query conditions were completely consistent in terms of text content, visual elements, layout structure, and semantic relationships. This grading standard provided a clear benchmark for evaluating retrieval performance, ensuring the consistency and reliability of evaluation results.

In the experimental parameter settings, the batch size of model training was 16, the number of iterations in the step-by-step pre-training stage was 100, and the learning rate was 0.0001; the number of iterations in the end-to-end fine-tuning stage was 50, and the learning rate was 0.00005; the optimizer used AdamW, the weight decay coefficient was 0.01, the temperature parameter was 0.07, and the margin parameter was 0.2; the FAISS vector database used IVF_FLAT index, the number of cluster centers was 100, and the number of neighboring cluster centers searched during query was 10. The marginal parameters were determined by comparing the retrieval performance under different boundary parameters (0.1, 0.2, 0.3, 0.5) on the validation set. The results showed that when the boundary parameter was 0.2, the model achieved the best Top-1 accuracy and average accuracy mean, which were 83.5% and 88.1%, respectively; When the boundary parameter was too small (such as 0.1), the discrimination between positive and negative samples was insufficient, resulting in a decrease in retrieval accuracy; When the boundary parameters were too large (such as 0.5), the difficulty of model training increased, the convergence speed slowed down, and underfitting was prone to occur. Therefore, selecting 0.2 as the equilibrium point for boundary parameters ensured effective differentiation between positive and negative samples while maintaining stable training of the model. The cluster number configuration was determined based on pre-experiments on the validation set: if the number of cluster centers was too small (such as 50), it would lead to insufficient discrimination in feature distribution and reduce the retrieval recall rate. If there was too much (such as 150), redundant computation would be introduced, increasing the retrieval delay. Experiments showed that, under the current dataset scale and feature distribution, when the number of cluster centers was 100 and the number of query clusters was 10, it was possible to maintain a high retrieval accuracy while stably controlling the response time of a single retrieval within the target range, achieving the best trade-off between retrieval efficiency and effect. The evaluation indicators used were Top-1 accuracy, Top-5 accuracy, Top-10 accuracy, mean average precision (mAP), F1 value, and retrieval response time. The loss weights for scene graph generation, cross modal fusion, and retrieval matching were set to 0.5, 0.3, and 0.2, respectively. This weight combination was determined through grid search optimization on the validation set: the search range was from 0.1 to 0.7 for each weight, with a step size of 0.1, for a total of 343 weight combinations. The experimental results showed that when the loss weight of scene graph generation was too high (such as 0.7 or above), the model focused too much on element detection and relationship prediction tasks, resulting in insufficient cross modal feature alignment ability, and the average accuracy on the validation set decreased by about 2.5%; When the weight of the retrieval matching loss was too high (such as 0.4 or above), the model was prone to overfitting. Although the retrieval matching loss decreased rapidly, the generalization ability weakened, and the Top-1 accuracy on the validation set fluctuated significantly. By comprehensively comparing the average accuracy of each weight combination with the Top-1 accuracy, 0.5:0.3:0.2 was determined as the optimal weight configuration. At this point, the average accuracy of the model on the validation set reached 88.1%, the Top-1 accuracy reached 83.5%, and the training process converged stably. This weight setting ensured the dominant position of the scene graph generation module as the basic feature extraction task, while also taking into account the optimization requirements of cross modal feature alignment and retrieval matching tasks, achieving an effective balance of multi task learning objectives.

To determine the optimal configuration of the number of cluster centers and query neighborhoods in the FAISS vector index, sensitivity analysis was conducted on the validation set. The number of cluster centers is set to 50, 100, and 150, respectively, and the number of query neighborhoods is set to 5, 10, and 20, respectively. The results are shown in Table 1. It can be observed that when the number of cluster centers is 50, the discriminative power of feature distribution is insufficient. Although the response time is short, the Top-1 accuracy is relatively low; When the number of cluster centers increases to 100, the accuracy significantly improves while the response time remains within an acceptable range; When it continues to increase to 150, the accuracy improvement tends to saturate, while the response time increases significantly. In terms of the number of query neighborhoods, when the number of query neighborhoods increases from 5 to 10, the accuracy steadily improves, but the increase in response time is limited; When further increased to 20, the accuracy improvement was weak, but the response time was significantly prolonged. Taking into account both retrieval accuracy and efficiency, this study selected a parameter configuration of 100 cluster centers and 10 query neighborhoods as the FAISS index. This configuration maintains high retrieval accuracy while controlling the response time of a single retrieval to 52.6ms, meeting the real-time retrieval needs of large-scale archives. The results of parameter sensitivity analysis have been shown in Table 1.

**Table 1:** Parameter sensitivity analysis.

Number of Cluster Centers	Query the number of neighborhoods	Top-1 accuracy (%)	Single search response time (ms)
50	5	81.6	46.2
	10	82.4	49.5
	20	82.7	55.8
100	5	83.1	50.3
	10	83.5	52.6
	20	83.6	59.4
150	5	83.2	54.7
	10	83.5	58.2
	20	83.7	65.1

### 3.2. Analysis of ablation experiment results

**Fig 7:**
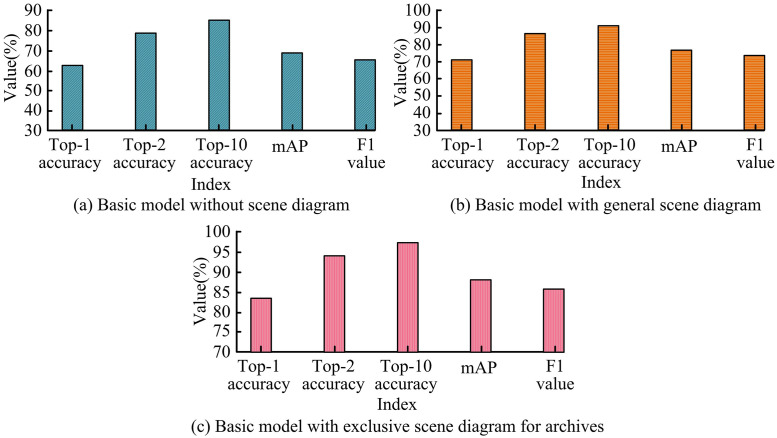
Ablation experiment results of the archive-specific scene graph generation module.

To verify the effectiveness of each core module of the model, ablation experiments were conducted on the test set. The ablation test results for the archive-specific scene graph generation module are shown in Fig 7. Fig 7(a) shows the base model without a scene graph, Fig 7(b) shows the base model with a general scene graph, and Fig 7(c) shows the base model with an archive-specific scene graph. Compared to the base model without a scene graph, the addition of the general scene graph significantly improved all model metrics, with Top-1 accuracy increasing by 9.2% and mAP increasing by 8.5%, demonstrating that the scene graph provided a visual semantic foundation for retrieval. Substituting the archive-specific scene graph further significantly improved metrics, with Top-1 accuracy increasing by 12.2% and mAP increasing by 11.4%. This was because the archive-specific scene graph optimized the detection and relationship definition of archival elements, more accurately capturing the visual semantic characteristics of archives, validating the necessity of the archive-specific scene graph generation module.

**Fig 8:**
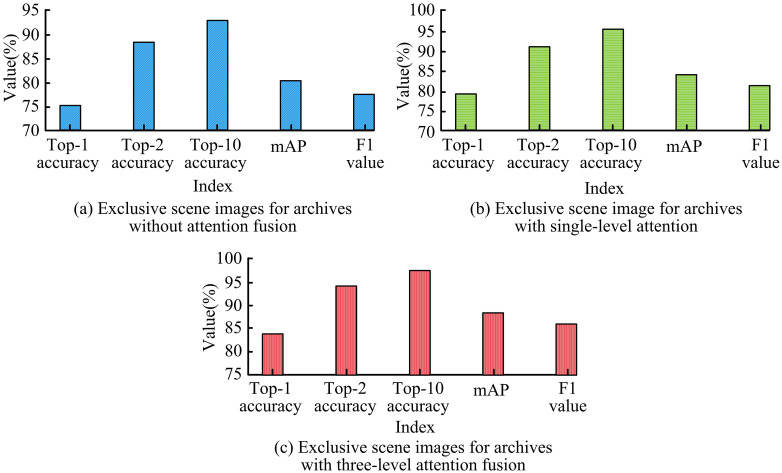
Ablation experiment results of three-level attention fusion module.

The ablation experiment results of the three-level attention fusion module are shown in Fig 8. Fig 8(a) shows the scene-specific scene graph without attention fusion, Fig 8(b) shows the archive-specific scene graph with single-level attention, and Fig 8(c) shows the archive-specific scene graph with three-level attention fusion. The results showed that without attention fusion, the model metrics were low, relying only on a simple concatenation of scene graph features and text features for retrieval. After adding single-level scene graph attention, the Top-1 accuracy improved by 4.2% and the mAP improves by 3.7%, demonstrating that attention could enhance key visual features. After adopting three-level attention fusion, all metrics reached optimal levels, with Top-1 accuracy improving by 8.5% and mAP improving by 7.8% compared to no attention fusion. This was because the three-level attention mechanism achieved precise alignment of visual semantics with linguistic semantics, effectively integrating the layout information of the scene graph with the semantic information of the text, verifying the effectiveness of the three-level attention fusion module.

Table 2 shows the results of ablation experiments using scene graph structure matching optimization. The study repeated the experiment for 5 times to assess each indicator. After introducing scene graph structure matching optimization, the retrieval accuracy of the model was significantly improved. The Top-1 accuracy increased from 80.1% ± 0.42% to 83.7% ± 0.38%, and the average accuracy increased from 85.6% ± 0.45% to 88.3% ± 0.41%. Through paired t-test verification, the above indicators showed statistically significant improvements (*p* < 0.01). Structural matching optimization effectively distinguished archive images with similar features but different structures by fusing logical information of nodes and relationships, thereby improving retrieval accuracy. Although the retrieval response time increased by 4.3ms, it still remained within 60ms, meeting the real-time requirements of archive retrieval. This result indicated that scene graph structure matching optimization significantly improved the retrieval performance of the model at an acceptable response time cost, verifying its effectiveness and necessity in fine-grained retrieval.

**Table 2:** Ablation experiment results of scene graph structure matching optimization.

Index	Third level attention fusion+unstructured matching	Three level attention fusion+scene graph structure matching
Top-1 accuracy (%)	80.1 ± 0.42	83.7 ± 0.38
Top-5 accuracy (%)	92.5 ± 0.31	94.2 ± 0.29
Top-10 accuracy (%)	96.2 ± 0.24	97.5 ± 0.22
mAP（%）	85.6 ± 0.455	88.3 ± 0.41
F1 value (%)	82.9 ± 0.51	85.9 ± 0.47
Search response time (ms)	48.3 ± 1.24	52.6 ± 1.31

In addition, to verify the effectiveness of the noise classification method, 2000 noisy archive images were randomly selected from the validation set for comparison between manual annotation and model classification results. Two strategies, random denoising (using median filtering without distinguishing noise types) and classification guided denoising (using this model method), were used for comparative experiments. The results are shown in Table 3. It can be seen that the classification guided denoising strategy improves the recall rate of text detection by 5.4% compared to the random denoising strategy, and the Top-1 accuracy and mAP increase by 3.2% and 3.1%, respectively. This indicates that accurate noise classification can adopt adaptive filtering methods for different types of noise, thereby more effectively restoring image quality and improving the performance of downstream text detection and retrieval tasks. In actual archival images, noise types often appear mixed. At this time, the system will prioritize salt and pepper noise detection and median filtering, and then perform Gaussian noise judgment and Gaussian filtering on the remaining noise to form a cascaded denoising process. This design ensures processing efficiency while enhancing denoising robustness in complex noisy scenes.

**Table 3:** Comparison of effectiveness results of noise classification methods.

Denoising strategy	Noise classification accuracy (%)	Text detection recall rate (%)	Top-1 accuracy (%)	mAP (%)
Random denoising	/	86.3	80.5	85.2
Classification guided denoising	94.2	91.7	83.7	88.3

To verify the rationality of the feature concatenation method, this study compared it with two mainstream relational inference methods on the validation set, namely the Relationship Graph Convolutional Network (R-GCN) and the Transformer based Relationship Inference Module (RelTransformer). The results are shown in Table 4. It can be found that the feature stitching method has a difference of no more than 0.3% in Top-1 accuracy and mAP compared to R-GCN and RelTransformer, and the retrieval accuracy of the three methods is basically the same. However, in terms of retrieval efficiency, the single response time of the feature stitching method is 52.6ms, while R-GCN and RelTransformer increase to 71.2ms and 89.5ms, respectively, which are 35.4% and 70.2% higher than the feature stitching method. This efficiency difference will be significantly amplified in large-scale archive retrieval scenarios, directly affecting the real-time response capability of the system. In addition, the relationship types in the archive scene diagram have highly structured and finite characteristics. According to the annotation statistics of the dataset, the relationships between archive elements can be clearly classified into four types: inclusion relationships, location relationships, association relationships, and membership relationships. Each type of relationship has clear boundaries and low ambiguity in semantics. In such a highly standardized semantic space, feature concatenation and multi classification through fully connected layers are sufficient to fully capture the association patterns between node pairs. The introduction of more complex inference modules brings extremely limited accuracy gains, but at the cost of significant efficiency losses. Therefore, the study chose feature concatenation as the basic method for relationship prediction, which meets the efficient retrieval needs of large-scale archives while ensuring retrieval accuracy, achieving the best balance between accuracy and efficiency.

**Table 4:** Comparison of results from different feature splicing methods.

Relational reasoning method	Top-1 accuracy (%)	mAP (%)	Single search response time (ms)
Feature concatenation (in this study)	83.5	88.1	52.6
R-GCN	83.7	88.3	71.2
RelTransformer	83.8	88.4	89.5

### 3.3. Comparative experimental results analysis

The comparative experiment selected four mainstream archival text image retrieval models as comparison objects, namely: (1) Optical Character Recognition + Term Frequency-Inverse Document Frequency (OCR + TF-IDF) model; (2) Scene Graph + Convolutional Neural Network (SceneGraph+CNN) model; (3) Contrastive Language-Image Pre-training (CLIP) model; and (4) Archive Retrieval Network (ARNet) model [27–29]. The comparison of retrieval accuracy between different models on the test set and a city level archive dataset in East China is shown in Table 5. The proposed model outperformed the comparison models in all retrieval accuracy indicators. Compared with the traditional OCR + TF-IDF model, the Top-1 accuracy as improved by 25.1% and the mAP is improved by 23.1%. This was because the proposed model integrated visual semantics and text semantics, avoiding retrieval failure caused by OCR recognition errors. Compared to the SceneGraph+CNN model, the proposed model achieved a 14.5% improvement in Top-1 accuracy and a 13.5% improvement in mAP, demonstrating the advantages of combining the archival-specific scene graph with cross-modal attention. Compared to the CLIP model, the proposed model achieved a 7.2% improvement in Top-1 accuracy and a 7.0% improvement in mAP. This was because CLIP lacked optimization for archival elements and was less robust to occlusions such as small text blocks and official seals. Compared to the ARNet model, the proposed model achieved a 3.4% improvement in Top-1 accuracy and a 3.2% improvement in mAP, thanks to its optimized scene graph structure matching, which allowed it to more accurately capture the fine-grained features of archival documents. On a city level archive dataset in East China, this model also achieved better retrieval performance than the comparison model. Specifically, the Top-1 accuracy of this model is 82.2%, which is 27.4%, 17.1%, 9.8%, and 6.4% higher than OCR + TF-IDF, SceneGraph+CNN, CLIP, and ARNet, respectively; The mAP is 85.7%, significantly higher than other models, indicating its good cross dataset generalization ability. Compared to the test set, the Top-1 accuracy of our model on this dataset decreased by only 1.5%, while the CLIP and ARNet models showed a decrease of 4.1% and 4.5%, respectively. This further demonstrates that our model has stronger robustness when facing different archive sources and image quality fluctuations. This is because the proposed model effectively enhances its fine-grained discrimination ability by introducing scene graph structure matching as a sorting optimization term, while domain adaptive design significantly enhances the model’s ability to model archive structures.

**Table 5:** Retrieval accuracy results of different models on the test set and a city level archive dataset in East China.

Dataset	Model	Top-1 accuracy (%)	Top-5 accuracy (%)	Top-10 accuracy (%)	mAP（%）	F1 value (%)
Test set	OCR + TF-IDF	58.6	75.3	82.1	65.2	62.4
	SceneGraph+CNN	69.2	84.5	90.3	74.8	72.1
	CLIP	76.5	89.8	94.6	81.3	78.7
	ARNet	80.3	92.1	96.4	85.1	82.6
	Proposed model	83.7	94.2	97.5	88.3	85.9
Dataset of a municipal level archive in East China	OCR + TF-IDF	54.8	71.2	78.6	61.3	58.6
	SceneGraph+CNN	65.1	80.3	86.3	70.2	67.5
	CLIP	72.4	86.1	90.1	77.3	74.9
	ARNet	75.8	88.4	92.5	80.5	78.2
	Proposed model	82.2	92.3	94.3	85.7	82.4

**Fig 9:**
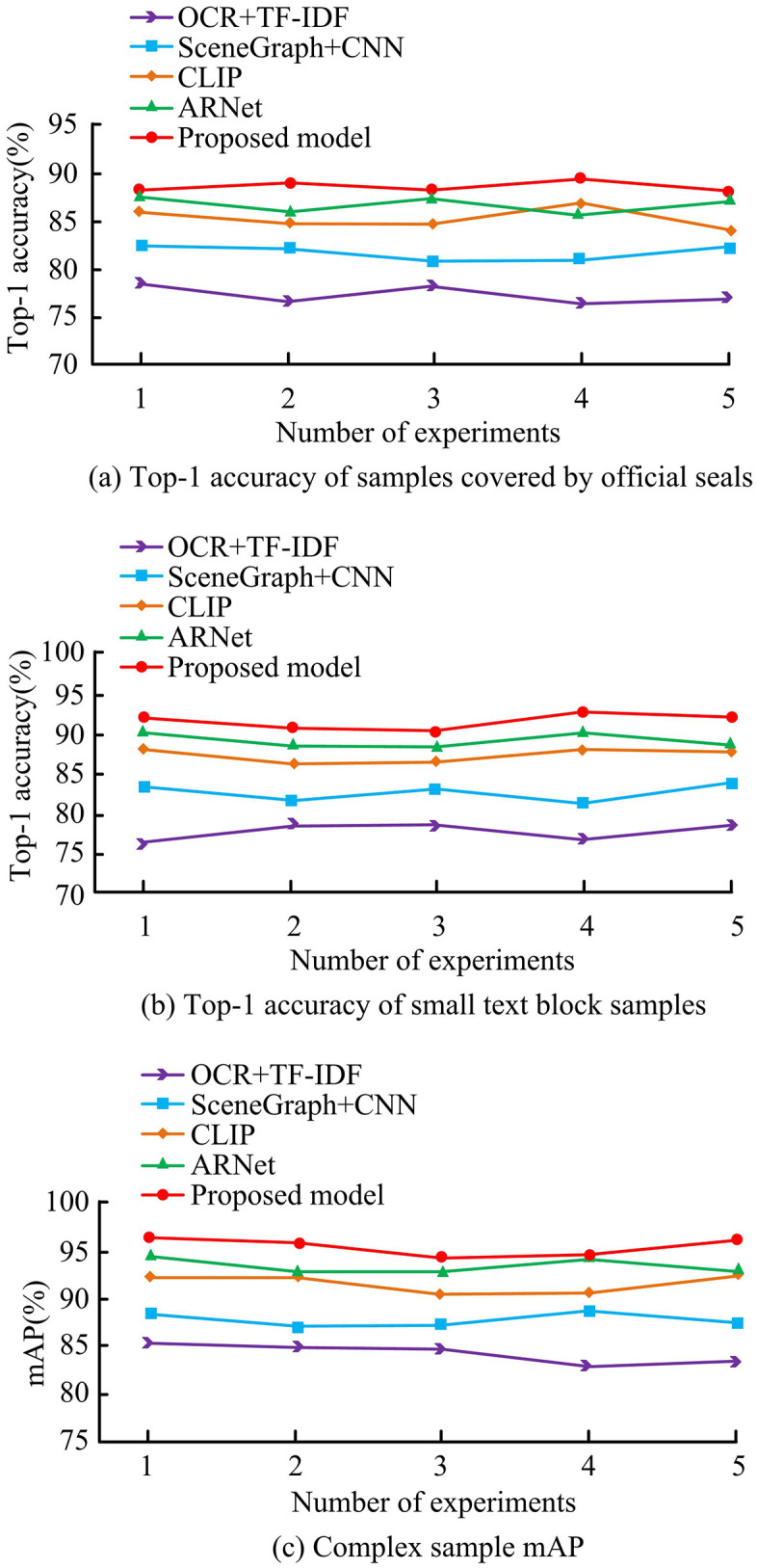
Comparison of retrieval performance of different models on complex archival images.

Fig 9 shows a comparison of the retrieval performance of different models on complex archival images. Fig 9(a) shows the Top-1 accuracy for the official seal occlusion sample, Fig 9(b) shows the Top-1 accuracy for the small text block sample, and Fig 9(c) shows the mAP for the complex sample. The results showed that the proposed model had a more pronounced advantage on complex samples. On the official seal occlusion sample, the proposed model’s Top-1 accuracy improved by 6.1% over ARNet and 11.2% over CLIP. On the small text block sample, the proposed model achieved a Top-1 accuracy of 76.8%, a 6.5% improvement over ARNet and 11.6% over CLIP. On the complex sample, the proposed model achieved a 5.7% improvement in mAP over ARNet. This was because the proposed model incorporated archival element priors into Faster R-CNN, optimizing small element detection accuracy. Furthermore, the proposed model captured the semantic associations between occluded elements and other elements through scene graph relationships, improving retrieval robustness in complex scenes.

**Fig 10:**
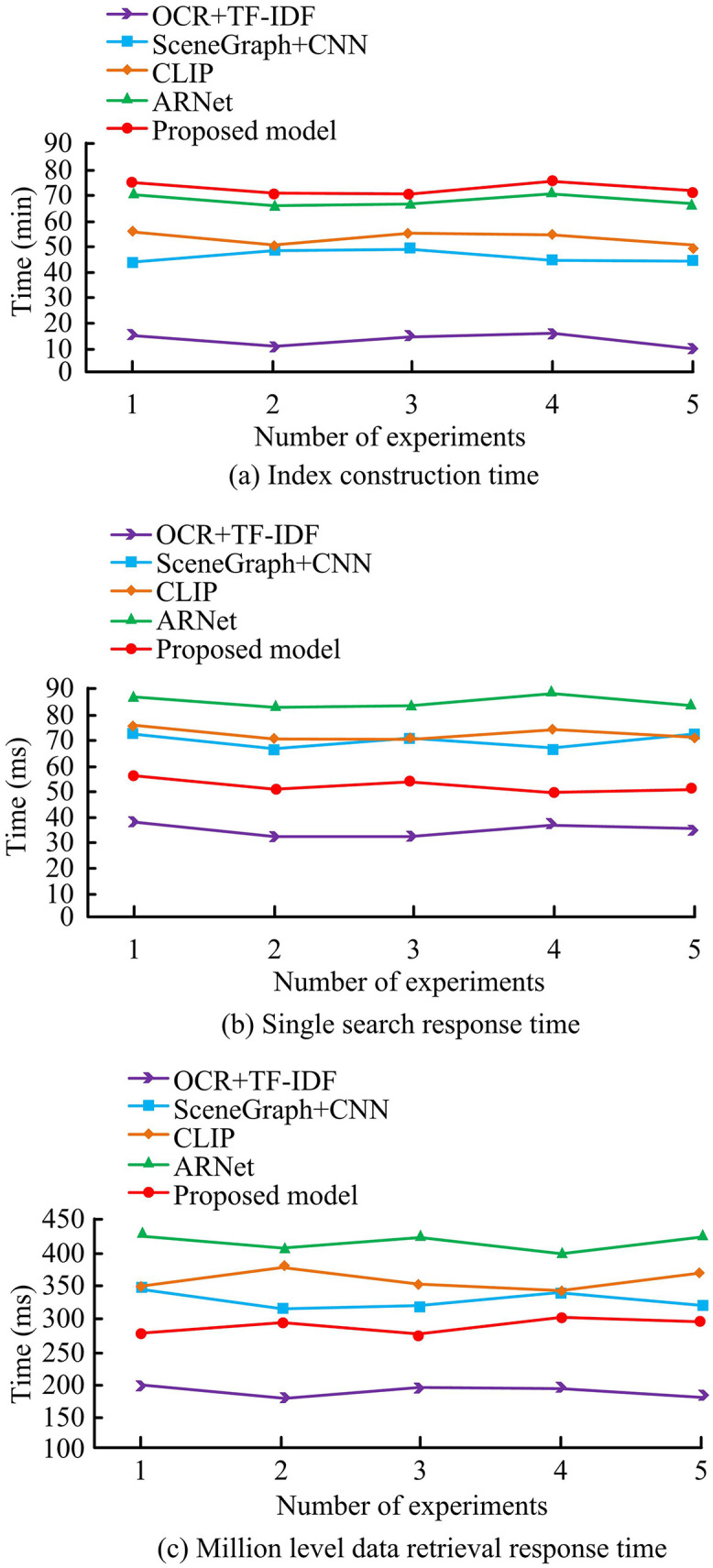
Comparison of retrieval efficiency of different models.

Fig 10 compares the retrieval efficiency of different models. Fig 10(a) shows the index construction time. The average index construction time for the proposed model was 72.6 minutes, slightly longer than other models due to the need to build a scene graph structure index. Fig 10(b) shows the single index response time. The average single retrieval response time for the proposed model was 52.6 milliseconds, outperforming SceneGraph+CNN, CLIP, and ARNet, and only slower than OCR + TF-IDF. This was due to FAISS’s IVF_FLAT index optimization and clustering search strategy. Fig 10(c) shows the index response time for millions of data points. The average response time for the proposed model was 289.4 milliseconds, which remained relatively low and met the retrieval efficiency requirements of large-scale archives, verifying the model’s balance between accuracy and efficiency.

### 3.4. Fine-grained retrieval case analysis

To further verify the fine-grained retrieval capability of the model, the experiment selected three typical archive retrieval scenarios for case analysis, namely: (1) official seal obscured text retrieval (querying for title text with official seal obscuration); (2) small text block retrieval (querying for archives with numbered small text blocks); and (3) multi-element association retrieval (querying for archives with specific tables + subordinate numbers). The feature similarity reflects the degree of matching between the query and the candidate archive in both visual and textual dimensions, with higher values indicating closer proximity in key elements, semantic content, and cross modal associations. The structural similarity was calculated based on the consistency of nodes and relationships in the scene graph, as shown in Equation (11), reflecting the degree of matching between the image layout structure and semantic logic.

The results of the official seal obscured text retrieval case are shown in Table 6. The query conditions were that the title contained the 2020 annual financial audit report and the official seal obscured the right 1/3 area of the title. The results showed that in the scenario where the official seal obscured the title, the proposed model could accurately identify the target file (A00125) and rank it first, with a final score of 91.5%, which was higher than other candidate files. Although A00512 had a high text feature similarity, its structural similarity was low due to the lack of official seal obscuration, and it was finally ranked third. A00943 had a high structural similarity, but a low text feature similarity, and was finally ranked fifth. This showed that the model could effectively distinguish files with similar texts but different occlusion structures through the weighted fusion of feature similarity and structural similarity, verifying the model’s fine-grained retrieval capability for official seal occlusion scenarios.

**Table 6:** Official seal obscured text search case results.

Candidate archive ID	Title text	The situation of the official seal being obstructed	Feature similarity (%)	Structural similarity (%)	Final score (%)
A00125	Financial audit report for 2020	Cover the right one-third of the title	92.3	89.5	91.5
A00368	Financial summary report for 2020	Cover the right quarter of the title	85.6	82.1	84.2
A00512	Financial audit report for 2019	Unobstructed	88.4	76.3	83.5
A00789	Personnel audit report for 2020	Cover the left one-third of the title	82.7	80.5	81.8
A00943	Financial audit report for 2021	Cover the right one-third of the title	86.2	88.7	87.3

**Fig 11:**
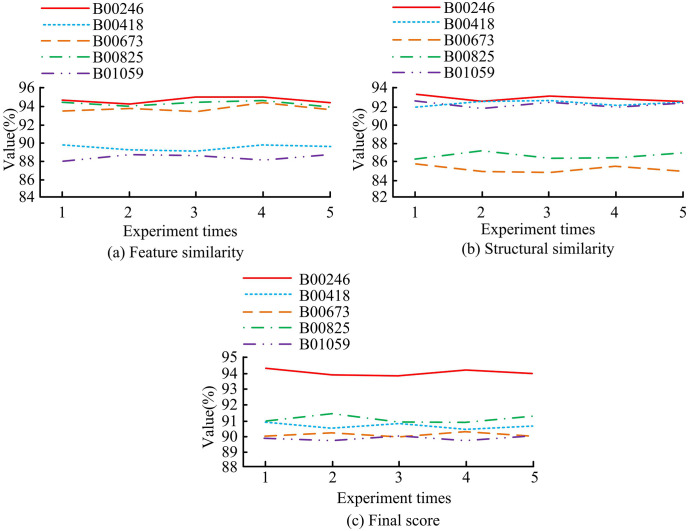
Small text block search case results.

The results of a small text block retrieval case are shown in Fig 11. The query condition was the number “Document Character (2023) No. 15,” the text block size was 120 × 80 pixels, and it was located on the left side of the image. Fig 11(a) shows the feature similarity, Fig 11(b) shows the structural similarity, and Fig 11(c) shows the final score. The results showed that for small text block retrieval, the proposed model accurately matched the target file B00246, achieving the highest final score of 94.0%. B00825 had a text feature similarity of 94.2%, close to the target file, but its structural similarity was lower due to the number being on the right, resulting in fourth place. B00673 had the correct number and position, but the text block size was inconsistent, resulting in lower structural similarity, resulting in third place. B00418 and B01059 had lower feature similarity due to differences in number and text. This demonstrated that the model, through small element detection optimization and structural matching in the file-specific scene graph, could capture fine-grained features such as the size and position of small text blocks, enabling accurate retrieval.

**Fig 12:**
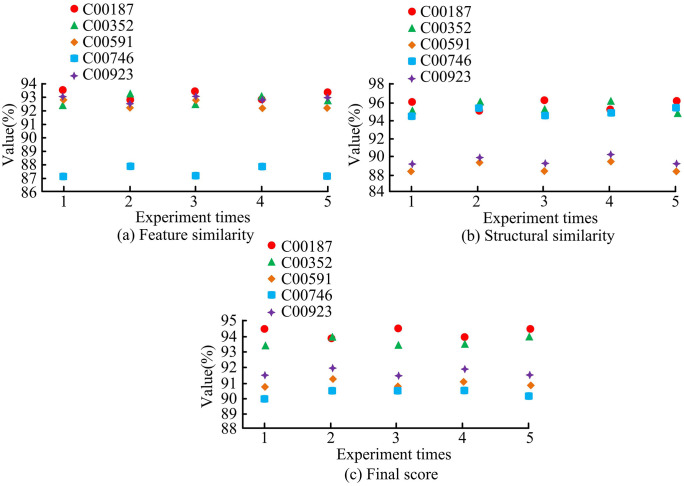
Multi-element association search case results.

The results of a multi-element association retrieval case are shown in Fig 12. The query condition was a combination of a fixed asset detailed table (containing 10 rows and 5 columns) and the subordinate number Shezi (2023) 08, with the number located above the table. Fig 12(a), 12(b), and 12(c) show the feature similarity, structural similarity, and final score, respectively. The results showed that in the multi-element association retrieval scenario, the proposed model accurately identified the target file C00187, achieving the highest final score of 94.2%. C00923 had high text feature similarity between the table and the number, but its structural similarity was low due to the number being located below the table, resulting in a final ranking of fifth. C00591 had the correct table name and number, but the table specifications did not match, resulting in a low structural similarity and a final ranking of third. C00746 had low feature similarity due to a different table name, resulting in a final ranking of fourth. This demonstrated that the model, through the relationship definition (subordinate relationship between table and number) and structural matching in the scene graph, could capture the association features between multiple elements, enabling fine-grained retrieval with multi-element collaboration and meeting the needs of complex archive retrieval.

## 4. Discussion

The research results fully demonstrate the effectiveness of the archive-specific scene graph and three-level attention fusion architecture in fine-grained retrieval. Its core value lies in overcoming the limitations of traditional archive retrieval, which relies on single text or visual features. Mechanistically, the archive-specific scene graph, through customized node definition and prior box optimization, precisely addresses the industry pain points of fuzzy detection of small text blocks and loss of semantic meaning due to occlusion by official seals. The three-level attention mechanism, through GAT weighting of key nodes in the scene graph, BERT reinforcement of text keywords, and cross-modal similarity alignment, achieved a deep coupling of visual layout and linguistic semantics. This was the fundamental reason why the three-level attention fusion architecture achieved a 7.8% improvement in mAP compared to the non-attention scheme in ablation experiments. Furthermore, the weighting strategy for feature and structural similarity in the retrieval and matching phase effectively distinguishes archived with similar text but different structures, further demonstrating the model’s ability to capture fine-grained archival features.

Compared with the existing mainstream models, the advantages of this research are reflected in three aspects: First, the Top-1 accuracy was improved by 25.1% compared with the OCR + TF-IDF model, avoiding the retrieval failure caused by OCR recognition errors, and proving the robustness of cross-modal fusion to text noise; second, the mAP was improved by 13.5% compared with the general SceneGraph+CNN model, highlighting the adaptability of the archive-specific scene graph to domain characteristics. The general scene graph did not take into account the grid line features of the archive table and the position regularity of the official seal, making it difficult to capture the unique logical relationship of the archive [30]. Thirdly, compared with advanced models such as CLIP and ARNet, the proposed model maintained an accuracy advantage of 3% −7%. Specifically, while ARNet performs effectively in general archive retrieval tasks, it does not explicitly model the logical relationships between elements, which limits its ability to distinguish archives that have different structures yet remain visually similar. In contrast, the proposed model utilizes these structured semantics to improve fine-grained retrieval performance, especially in scenarios involving sealed occlusion and small text blocks, with an improvement of over 6%.

This study has two limitations: First, the dataset came from a single provincial archive. While the archive types covered were representative, they did not cover special scenarios such as historical handwritten archives and multilingual archives, requiring further verification of the model’s universality. Second, while the index construction time met the requirements of a million-level archive, it is still longer than that of OCR + TF-IDF, and the indexing efficiency of large-scale archives needs to be optimized. Future research could be advanced in two ways: first, expanding the dataset to include handwritten and multilingual archives, optimizing the texture features of scene graph nodes and the text recognition module; and second, introducing quantitative indexing technology to shorten index construction time while ensuring accuracy.

## 5. Conclusion

Aiming at the demand for visual-text dual feature fusion of archival text images, the study constructed a five-order fine-grained retrieval model consisting of input layer, preprocessing layer, scene graph generation layer, attention fusion layer, and retrieval matching layer. The archive-specific scene graph generation module effectively improved the ability to capture domain features. The experimental results showed that the model achieved a Top-1 accuracy of 83.7% and an mAP of 88.3% on the test set, demonstrating excellent fine-grained retrieval performance in typical scenarios such as seal occlusion, small text blocks, and multi-element association. The archive specific scene graph generation module effectively improved the ability to capture domain features. Through customized node definitions, prior box optimization, and structural simplification, the accuracy increased by 12.2% compared to the Top-1 of general scene graphs, solving the problem of detecting small text blocks and seal occlusions. Although the model achieved good performance in retrieval accuracy and response speed, there are still certain limitations. Although the average indexing time of the model meets the requirements of building millions of archives, the indexing efficiency in large-scale dynamic update scenarios needs to be optimized. Further optimization can be carried out in future research. In terms of handwritten archives, a multi lingual pre trained model based on convolutional recurrent networks and linked temporal classification can be introduced as the text encoding basis. Language identification attributes can be introduced in scene graph nodes to construct a cross lingual semantic alignment module. Finally, the inverted index compression technique based on product quantization can shorten the index construction time while ensuring retrieval accuracy.
