# Supplementary material for: Construction of a fine-grained retrieval model for archival text-image based on the integration of scene graph generation and attention mechanism
Source: PLoS One. 2026 Jul 24;21(7):e0353505. doi: 10.1371/journal.pone.0353505 (PMC13399324; doi:10.1371/journal.pone.0353505)
Supplement: Attachment — Submitted filename: Response to comments.doc [file pone.0353505.s002.doc]

Reviewer #1:

This study proposes a fine-grained retrieval model for archival text-images by integrating archive-specific scene graph generation with a three-level attention fusion mechanism. The model consists of five layers: input, preprocessing, scene graph generation, attention fusion, and retrieval matching.

**The core innovation lies in:**

Designing an archive-specific scene graph module to better detect and represent key archival elements (e.g., seals, small text blocks, tables, serial numbers).

Introducing a three-level attention mechanism (scene graph attention, text attention using BERT, and cross-modal attention) to align visual and textual features.

Combining feature similarity and structural graph matching to improve retrieval ranking.

Experiments conducted on a dataset of 50,000 archival images demonstrate that the proposed model achieves 83.7% Top-1 accuracy and 88.3% mAP, outperforming OCR+TF-IDF, SceneGraph+CNN, CLIP, and ARNet models. The model also shows robustness in complex scenarios such as official seal occlusion and small text blocks, while maintaining acceptable retrieval efficiency.

Strengths

+Clear research motivation: The paper clearly addresses the practical challenges of archival retrieval, especially noise, seal occlusion, and small text blocks.

+Well-structured methodology: The five-layer architecture is logically organized and systematically described.

+Domain-specific innovation: The archive-specific scene graph (custom node definitions and prior box optimization) is a meaningful domain adaptation.

+Comprehensive technical design: Includes detailed formulations for graph construction, attention mechanisms, similarity computation, and multi-task loss.

+Strong experimental validation:

++Large real-world dataset (50,000 images).

++Multiple evaluation metrics (Top-1/5/10, mAP, F1, response time).

++Ablation studies validating each core module.

++Comparisons with strong baselines (CLIP, ARNet).

+Case study analysis: Detailed fine-grained retrieval cases (seal occlusion, small text block, multi-element association) enhance practical relevance.

+Balanced accuracy and efficiency analysis: Includes index construction time and large-scale retrieval response time.

+Discussion and limitations provided: The authors explicitly acknowledge dataset and indexing limitations.

Reply: Thank you very much for your positive evaluation for our work.

Weaknesses

-Writing clarity and language quality: Some grammatical inconsistencies and awkward phrasing reduce readability. The manuscript would benefit from professional language editing.

Reply: Thank you very much for your comments. The language has been improved by scholars who have published high level English papers.

-Overly dense methodological section: The large number of equations and technical details may obscure the main conceptual contributions.

Reply: Thank you very much for your comments. The detailed process, boundary parameter determination, and clustering quantity configuration of the preprocessing layer have been streamlined. Please see the revisions as follows:

Redundant edges include edges connecting blank areas and edges with relationship strength below a threshold and non core relationships, which are pruned [23].

The preprocessing layer adopts a four step process, including noise classification, targeted denoising, text detection, and structured recognition. Firstly, it distinguishes between salt and pepper noise and Gaussian noise through a noise statistical model, and uses median filtering and Gaussian filtering respectively for denoising. The noise statistical model is based on the local grayscale statistical characteristics of the image: salt and pepper noise is manifested as randomly appearing extremely bright or dark pixels, with grayscale values close to 0 or 255, and significant differences in grayscale from neighboring pixels; Gaussian noise is manifested as random fluctuations in the normal distribution of grayscale values, with large local variances but relatively continuous grayscale value ranges. When making specific judgments, calculate the grayscale range and variance within the local window (3 × 3 pixels) of the image. If the range is large and there are extreme grayscale values (<5 or>250), it is judged as salt and pepper noise; If the variance is large but there are no extreme grayscale values, it is judged as Gaussian noise. Subsequently, EAST is used to locate the text block area. Finally, text recognition is achieved through optimized Tesseract OCR, which outputs clean images and structured text, including three types of structured information: title, body, and number.

As shown in Figure 6, step-by-step pre-training is first performed with 100 iterations and a learning rate of 0.0001. The scene graph generation module (Faster R-CNN and GAT) is pre-trained using annotated archival images, while the text encoding module (BERT) is pre-trained on 100,000 archival texts. Subsequently, end-to-end fine-tuning is conducted with 50 iterations and a learning rate of 0.00005, combining all modules to optimize the overall loss function using the archival retrieval dataset.

-Limited dataset diversity: Data originate from a single provincial archive, which may limit generalizability.

Reply: Thank you for your feedback on the manuscript. Under your guidance, other datasets have been supplemented as required, as follows.

In addition, to evaluate the generalization ability of the research model, the study introduced a city level archive in East China, which contains 15000 archival graphic and textual images, covering document archives (9000), accounting archives (3500), and scientific and technological archives (2500). The image resolution range is from 1000 × 1400 pixels to 2000 × 2800 pixels, of which 32% are samples of official seal occlusion and 38% are samples of small text blocks.

On a city level archive dataset in East China, this model also achieved better retrieval performance than the comparison model. Specifically, the Top-1 accuracy of this model is 82.2%, which is 27.4%, 17.1%, 9.8%, and 6.4% higher than OCR+TF-IDF, SceneGraph+CNN, CLIP, and ARNet, respectively; The mAP is 85.7%, significantly higher than other models, indicating its good cross dataset generalization ability. Compared to the test set, the Top-1 accuracy of our model on this dataset decreased by only 1.5%, while the CLIP and ARNet models showed a decrease of 4.1% and 4.5%, respectively. This further demonstrates that our model has stronger robustness when facing different archive sources and image quality fluctuations. This is because the proposed model effectively enhances its fine-grained discrimination ability by introducing scene graph structure matching as a sorting optimization term, while domain adaptive design significantly enhances the model's ability to model archive structures.

Table 5 Retrieval accuracy results of different models on the test set and a city level archive dataset in East China

| Dataset | Model | Top-1 accuracy (%) | Top-5 accuracy (%) | Top-10 accuracy (%) | mAP（%） | F1 value (%) |
| --- | --- | --- | --- | --- | --- | --- |
| Test set | OCR+TF-IDF | 58.6 | 75.3 | 82.1 | 65.2 | 62.4 |
|  | SceneGraph+CNN | 69.2 | 84.5 | 90.3 | 74.8 | 72.1 |
|  | CLIP | 76.5 | 89.8 | 94.6 | 81.3 | 78.7 |
|  | ARNet | 80.3 | 92.1 | 96.4 | 85.1 | 82.6 |
|  | Proposed model | 83.7 | 94.2 | 97.5 | 88.3 | 85.9 |
| Dataset of a municipal level archive in East China | OCR+TF-IDF | 54.8 | 71.2 | 78.6 | 61.3 | 58.6 |
|  | SceneGraph+CNN | 65.1 | 80.3 | 86.3 | 70.2 | 67.5 |
|  | CLIP | 72.4 | 86.1 | 90.1 | 77.3 | 74.9 |
|  | ARNet | 75.8 | 88.4 | 92.5 | 80.5 | 78.2 |
|  | Proposed model | 82.2 | 92.3 | 94.3 | 85.7 | 82.4 |

-Recent references dominate: Many citations are from 2024–2025, and foundational works in scene graph generation and cross-modal retrieval could be cited more comprehensively.

Reply: Thank you very much for your comments. Due to the lack of relevant literature on scenario graph generation in 2024-2025, the study only supplemented literature related to cross modal retrieval. The specific modifications are as follows:

The weighted text features are shown in equation (7) [25].

The comparative experiment selected four mainstream archival text image retrieval models as comparison objects, namely: (1) Optical Character Recognition + Term Frequency-Inverse Document Frequency (OCR+TF-IDF) model; (2) Scene Graph + Convolutional Neural Network (SceneGraph+CNN) model; (3) Contrastive Language-Image Pre-training (CLIP) model; and (4) Archive Retrieval Network (ARNet) model [27-29].

The general scene graph did not take into account the grid line features of the archive table and the position regularity of the official seal, making it difficult to capture the unique logical relationship of the archive [30].

[4] J. Strader, N. Hughes, W. Chen, A. Speranzon and L. Carlone. "Indoor and Outdoor 3D Scene Graph Generation Via Language-Enabled Spatial Ontologies," IEEE ROBOT AUTOM LET, vol. 9, no. 6, pp. 4886-4893, June, 2024, DOI: 10.1109/LRA.2024.3384084.

[6] C. Lv, M. Qi, X. Li, Z. Yang and H. Ma. “Sgformer: Semantic graph transformer for point cloud-based 3d scene graph generation," AAAI, vol. 38, no. 5, pp. 4035-4043, February, 2024, DOI: 10.1609/aaai.v38i5.28197.

[25] J. Peng, X. Xiang, J. Qin and Y. Tan. "Dual-branch networks for privacy-preserving cross-modal retrieval in cloud computing," J. Supercomput., vol. 81, no. 1, pp. 1-21, January. 2025, DOI: 10.1007/s11227-024-06643-3.

[27] L. Yang, W. Zhang, Y. Miao, Y. Liang,X. Li,K. K. R. Choo and R. H. Deng. "Secure and efficient cross-modal retrieval over encrypted multimodal data," IEEE Trans. Comput., early access, vol. 74, no. 4, pp. 1405-1417, February, 2025, DOI: 10.1109/TC.2025.3525614.

[30] J. Deng, W. Ou, J. Gou, H/ Song, A. Wang and X. Xu. "Representation separation adversarial networks for cross-modal retrieval," Wirel. Netw., vol. 30, no. 5, pp. 3469-3481, May, 2024, DOI: 10.1007/s11276-020-02382-4.

-Hyperparameter justification is limited: Some parameter choices (e.g., weighting 0.7/0.3 in final score fusion) are not deeply analyzed.

Reply: Thank you very much for your comments. The in-depth analysis of parameter selection has been supplemented, as follows.

In the experimental parameter settings, the batch size of model training was 16, the number of iterations in the step-by-step pre-training stage was 100, and the learning rate was 0.0001; the number of iterations in the end-to-end fine-tuning stage was 50, and the learning rate was 0.00005; the optimizer used AdamW, the weight decay coefficient was 0.01, the temperature parameter was 0.07, and the margin parameter was 0.2; the FAISS vector database used IVF_FLAT index, the number of cluster centers was 100, and the number of neighboring cluster centers searched during query was 10. The marginal parameters are determined by comparing the retrieval performance under different boundary parameters (0.1, 0.2, 0.3, 0.5) on the validation set The results showed that when the boundary parameter was 0.2, the model achieved the best Top-1 accuracy and average accuracy mean, which were 83.5% and 88.1%, respectively; When the boundary parameter is too small (such as 0.1), the discrimination between positive and negative samples is insufficient, resulting in a decrease in retrieval accuracy; When the boundary parameters are too large (such as 0.5), the difficulty of model training increases, the convergence speed slows down, and underfitting is prone to occur. Therefore, selecting 0.2 as the equilibrium point for boundary parameters ensures effective differentiation between positive and negative samples while maintaining stable training of the model. The cluster number configuration is determined based on pre-experiments on the validation set: if the number of cluster centers is too small (such as 50), it will lead to insufficient discrimination in feature distribution and reduce the retrieval recall rate. If there is too much (such as 150), redundant computation will be introduced, increasing the retrieval delay. Experiments show that, under the current dataset scale and feature distribution, when the number of cluster centers is 100 and the number of query clusters is 10, it is possible to maintain a high retrieval accuracy while stably controlling the response time of a single retrieval within the target range, achieving the best trade-off between retrieval efficiency and effect.

-Figure presentation: Some figures are crowded and could be improved for clarity and visual consistency.

Reply: Thank you very much for your comments. The quality and layout of the Figures have been improved, please review again.

-Reproducibility details: Although dataset statistics are provided, public availability of data/code is not clearly specified in the manuscript.

Reply: Thank you very much for your comments. The public availability of data/code has been provided as follows:

**Data and code availability**

The "Minimal Data Set" file of the manuscript is available at: https://zenodo.org/uploads/20070215

The "Code" file of the manuscript is available at: https://zenodo.org/uploads/20070153.

Overall Recommendation

-The manuscript presents a technically solid and experimentally validated contribution to fine-grained archival retrieval. The integration of archive-specific scene graph modeling with multi-level attention fusion is meaningful and well-supported by experiments.

The identified weaknesses are primarily related to clarity, presentation, and minor methodological justification, rather than fundamental flaws in design or validation.

Recommendation: Minor revisions.

Suggested revisions:

Improve language quality and readability.

Reply: Thank you very much for your comments. The language has been improved by scholars who have published high level English papers.

Clarify parameter selection rationale (e.g., fusion weights).

Reply: Thank you very much for your comments. The parameter selection rationale has been supplemented, as follows:

Among them, the feature similarity weight is set to 0.7, and the structure similarity weight is set to 0.3. This weight combination is obtained through grid search optimization on the validation set. This weight setting reflects the dominant role of feature similarity in retrieval and matching. Structural similarity serves as a supplementary constraint, effectively enhancing the ability to distinguish archival images with similar visual features but significant structural differences.

In the experimental parameter settings, the batch size of model training was 16, the number of iterations in the step-by-step pre-training stage was 100, and the learning rate was 0.0001; the number of iterations in the end-to-end fine-tuning stage was 50, and the learning rate was 0.00005; the optimizer used AdamW, the weight decay coefficient was 0.01, the temperature parameter was 0.07, and the margin parameter was 0.2; the FAISS vector database used IVF_FLAT index, the number of cluster centers was 100, and the number of neighboring cluster centers searched during query was 10. The marginal parameters were determined by comparing the retrieval performance under different boundary parameters (0.1, 0.2, 0.3, 0.5) on the validation set. The results showed that when the boundary parameter was 0.2, the model achieved the best Top-1 accuracy and average accuracy mean, which were 83.5% and 88.1%, respectively; When the boundary parameter was too small (such as 0.1), the discrimination between positive and negative samples was insufficient, resulting in a decrease in retrieval accuracy; When the boundary parameters were too large (such as 0.5), the difficulty of model training increased, the convergence speed slowed down, and underfitting was prone to occur. Therefore, selecting 0.2 as the equilibrium point for boundary parameters ensured effective differentiation between positive and negative samples while maintaining stable training of the model. The cluster number configuration was determined based on pre-experiments on the validation set: if the number of cluster centers was too small (such as 50), it would lead to insufficient discrimination in feature distribution and reduce the retrieval recall rate. If there was too much (such as 150), redundant computation would be introduced, increasing the retrieval delay. Experiments showed that, under the current dataset scale and feature distribution, when the number of cluster centers was 100 and the number of query clusters was 10, it was possible to maintain a high retrieval accuracy while stably controlling the response time of a single retrieval within the target range, achieving the best trade-off between retrieval efficiency and effect.

Strengthen discussion on generalizability and dataset diversity.

Reply: Thank you for providing detailed revision suggestions for the manuscript. The discussion on generalizability and dataset diversity has been supplemented as follows.

In addition, to evaluate the generalization ability of the research model, the study introduced a city level archive in East China, which contains 15000 archival graphic and textual images, covering document archives (9000), accounting archives (3500), and scientific and technological archives (2500). The image resolution range is from 1000 × 1400 pixels to 2000 × 2800 pixels, of which 32% are samples of official seal occlusion and 38% are samples of small text blocks.

On a city level archive dataset in East China, this model also achieved better retrieval performance than the comparison model. Specifically, the Top-1 accuracy of this model is 82.2%, which is 27.4%, 17.1%, 9.8%, and 6.4% higher than OCR+TF-IDF, SceneGraph+CNN, CLIP, and ARNet, respectively; The mAP is 85.7%, significantly higher than other models, indicating its good cross dataset generalization ability. Compared to the test set, the Top-1 accuracy of our model on this dataset decreased by only 1.5%, while the CLIP and ARNet models showed a decrease of 4.1% and 4.5%, respectively. This further demonstrates that our model has stronger robustness when facing different archive sources and image quality fluctuations. This is because the proposed model effectively enhances its fine-grained discrimination ability by introducing scene graph structure matching as a sorting optimization term, while domain adaptive design significantly enhances the model's ability to model archive structures.

Table 5 Retrieval accuracy results of different models on the test set and a city level archive dataset in East China

| Dataset | Model | Top-1 accuracy (%) | Top-5 accuracy (%) | Top-10 accuracy (%) | mAP（%） | F1 value (%) |
| --- | --- | --- | --- | --- | --- | --- |
| Test set | OCR+TF-IDF | 58.6 | 75.3 | 82.1 | 65.2 | 62.4 |
|  | SceneGraph+CNN | 69.2 | 84.5 | 90.3 | 74.8 | 72.1 |
|  | CLIP | 76.5 | 89.8 | 94.6 | 81.3 | 78.7 |
|  | ARNet | 80.3 | 92.1 | 96.4 | 85.1 | 82.6 |
|  | Proposed model | 83.7 | 94.2 | 97.5 | 88.3 | 85.9 |
| Dataset of a municipal level archive in East China | OCR+TF-IDF | 54.8 | 71.2 | 78.6 | 61.3 | 58.6 |
|  | SceneGraph+CNN | 65.1 | 80.3 | 86.3 | 70.2 | 67.5 |
|  | CLIP | 72.4 | 86.1 | 90.1 | 77.3 | 74.9 |
|  | ARNet | 75.8 | 88.4 | 92.5 | 80.5 | 78.2 |
|  | Proposed model | 82.2 | 92.3 | 94.3 | 85.7 | 82.4 |

This study has two limitations: First, the dataset came from a single provincial archive. While the archive types covered were representative, they did not cover special scenarios such as historical handwritten archives and multilingual archives, requiring further verification of the model's universality. Second, while the index construction time met the requirements of a million-level archive, it is still longer than that of OCR+TF-IDF, and the indexing efficiency of large-scale archives needs to be optimized. Future research could be advanced in two ways: first, expanding the dataset to include handwritten and multilingual archives, optimizing the texture features of scene graph nodes and the text recognition module; and second, introducing quantitative indexing technology to shorten index construction time while ensuring accuracy.

Although the average indexing time of the model meets the requirements of building millions of archives, the indexing efficiency in large-scale dynamic update scenarios needs to be optimized. Further optimization can be carried out in future research. In terms of handwritten archives, a multi lingual pre trained model based on convolutional recurrent networks and linked temporal classification can be introduced as the text encoding basis. Language identification attributes can be introduced in scene graph nodes to construct a cross lingual semantic alignment module. Finally, the inverted index compression technique based on product quantization can shorten the index construction time while ensuring retrieval accuracy.

Improve figure clarity and formatting.

Reply: Thank you very much for your comments. The quality and layout of the Figures have been improved, please review again.

Clarify data/code availability for reproducibility.

Reply: Thank you very much for your comments. The repeatability of the data has been explained, as follows.

The experimental dataset came from the historical and modern archives of a provincial archives, containing a total of 50,000 archival text images, covering three categories: document archives (32,000 images), accounting archives (8,000 images), and scientific and technological archives (10,000 images). The image resolution ranged from 1200×1600 pixels to 2400×3200 pixels, and contained typical archival image problems such as salt and pepper noise (18%), Gaussian noise (22%), official seal occlusion (35%), and small text blocks (42%). The dataset was divided into a training set (35,000 images), a validation set (10,000 images), and a test set (5,000 images) in a ratio of 7:2:1. All images were annotated with node information (four categories: text blocks, official seals, tables, and blank areas, totaling 182,000 nodes) and relationship information (four categories: containment, location, association, and subordination, totaling 256,000 relationships). Structured text labels (title, body, number) and search similarity labels (levels 1-5, with level 1 being a complete mismatch and level 5 being a complete match) were also provided.

All images were annotated at multiple levels. At the node level, four core elements including text blocks, seals, tables, and blank areas were annotated, totaling 182000 nodes. On average, each image contains about 3.6 nodes, with text block nodes accounting for the highest proportion, about 62%. At the relational level, four types of semantic relationships were annotated, including relationships, orientation relationships, association relationships, and membership relationships, totaling 256000 relationships. On average, each image contains about 5.1 relationships, with orientation relationships and association relationships being the most common, accounting for 38% and 29% of the total correlation coefficients, respectively. The above annotation density ensures the integrity and semantic richness of scene graph construction. The retrieval similarity tags adopt a five level scoring system, and are independently annotated by three experts in the field of archives based on the correlation between query conditions and archive images. The final tags are based on the consensus of the majority or expert consultation results. The specific definitions of each level are as follows: Level 1 (completely unrelated) indicates that there is no correlation between the image content and the query conditions in terms of theme, elements, and structure, such as when querying the "2020 Financial Audit Report" and returning the "1985 Personnel Appointment and Removal Notice"; Level 2 (partially related) indicates that the image only has a very small number of common elements with the query criteria, but the overall theme and structure do not match, such as the query containing "seals" but returning images with only similar seals and no matching text; Level 3 (moderately correlated) indicates that the image matches the query criteria on some key elements, but there are significant differences in overall structure or semantic associations, such as matching table formats but not matching document numbers; Level 4 (highly correlated) indicates a high degree of match between the image and the query criteria in terms of primary elements and structure, with only minor differences in details such as title matching but slight offset in seal position; Level 5 (completely related) indicates that the image and query conditions are completely consistent in terms of text content, visual elements, layout structure, and semantic relationships. This grading standard provides a clear benchmark for evaluating retrieval performance, ensuring the consistency and reliability of evaluation results.

With these refinements, the paper would be suitable for publication.

Reply: Thank you very much for your kind recommendation. The paper has been revised according to your revies comments and suggestions, please review again.

Reviewer #2:

The introduction cites recent papers but does not critically engage with their limitations to clearly establish the unique contribution of this study.

Reply: Thank you very much for your comments. The limitations of the cited papers have been explained, plese see details as follows:

C. M. Lo et al. extracted high-level features by fine-tuning the deep learning architecture for medical image retrieval, and used three different annotated images for classification and query. The results showed that the classification accuracy of this method reached 94% [7]. However, the above-mentioned methods are highly dependent on domain-specific labeled data and do not take into account the semantic alignment issue between text and images, making it difficult to be transferred to multimodal and strongly structured scenarios such as archival text and images.

Yao et al. proposed a simple yet effective image-text retrieval method, called cross-modal interactive reasoning, for enhancing visual language pre-training. Extensive experiments have shown that the research method achieves 52 and 97.5 results on two widely used datasets compared to the state-of-the-art non-pre-trained methods, and it also outperforms several mainstream fine-tuned visual language pre-trained models [11]. Although cross-modal pre-trained models perform well in general retrieval tasks, they have the problem of semantic confusion in archival image retrieval, that is, the models have difficulty understanding the specific semantic logic of archives, such as the affiliation relationship between document numbers and main text, and the positional relationship between seals and titles. In addition, most historical image retrieval methods focus on visual content and do not fully integrate structured text information.

Most of the existing efficient retrieval methods focus on optimization at the system level, while they are still relatively weak at the semantic representation level. The domain adaptation and texture enhancement techniques used in remote sensing image retrieval can provide inspiration for element detection in archival images, but cross-modal alignment still needs to be combined with text semantics.

Therefore, the study proposes a fine-grained retrieval model for archival text images that integrates scene graph generation and attention mechanisms. This model adopts a five-layer architecture, including the input layer, preprocessing layer, scene graph generation layer, attention fusion layer and retrieval matching layer. It optimizes element detection by constructing a dedicated scene graph module for archives and designs a three-level attention mechanism to achieve deep integration of visual and text features. The innovation of the research is mainly reflected in the fact that, in response to the fine-grained retrieval needs of archival text images, a multi-stage architecture that integrates text and visual features is designed. The key elements and their relationships are accurately extracted through the archive-specific scene graph generation module, and the multi-dimensional attention mechanism is combined to achieve deep fusion of cross-modal features. Finally, the retrieval accuracy and efficiency are improved through a retrieval matching module that combines feature similarity and structural matching.

The description of "prior box" optimization lacks empirical data or analysis from the dataset to justify the chosen scales and aspect ratios.

Reply: Thank you very much for your comments. In the design of the prior box, the selection of scale and aspect ratio has been based on the statistical characteristics of the dataset, and the design basis and calculation formula have been clearly provided in the text. As shown in the ablation experiment results of Figure 7, using the archive specific scene graph (including optimized prior boxes) resulted in a 12.2% increase in Top-1 accuracy and an 11.4% increase in mAP compared to the general scene graph. This significant improvement indirectly proves the effectiveness of prior box optimization. If the scale and aspect ratio of the prior box are not designed properly, the archive specific scene graph module cannot bring such a significant performance improvement. The specific content is as follows:

The scales and aspect ratios of these prior boxes are designed based on the statistical characteristics of archive elements, with the scale calculation shown in equation (1) [18].

$s_{k}=s_{\min}+(s_{\max}-s_{\min})\cdot\frac{k-1}{m-1}$ (1)

In equation (1), $s_{k}$ is the scale (pixel) of the $k$th prior, representing the ratio of the edge length of the prior box to the edge length of the image feature map; $s_{\min}$ is the minimum prior box scale; $s_{\max}$ is the maximum prior box scale; $k$ is the prior box index; $m$ is the number of prior boxes.

The archive specific scene graph generation module effectively improves the ability to capture domain features. Through customized node definitions, prior box optimization, and structural simplification, the accuracy has increased by 12.2% compared to the Top-1 of general scene graphs, solving the problem of detecting small text blocks and seal occlusions.

The noise classification method in the preprocessing layer is mentioned but the specific statistical model and its accuracy are not explained.

Reply: Thank you for providing detailed revision suggestions for the manuscript. The specific statistical model and its accuracy have been supplemented as follows.

Firstly, it distinguishes between salt and pepper noise and Gaussian noise through a noise statistical model, and uses median filtering and Gaussian filtering respectively for denoising. The noise statistical model is based on the local grayscale statistical characteristics of the image: salt and pepper noise is manifested as randomly appearing extremely bright or dark pixels, with grayscale values close to 0 or 255, and significant differences in grayscale from neighboring pixels; Gaussian noise is manifested as random fluctuations in the normal distribution of grayscale values, with large local variances but relatively continuous grayscale value ranges. When making specific judgments, calculate the grayscale range and variance within the local window (3 × 3 pixels) of the image. If the range is large and there are extreme grayscale values (<5 or>250), it is judged as salt and pepper noise; If the variance is large but there are no extreme grayscale values, it is judged as Gaussian noise.

In addition, to verify the effectiveness of the noise classification method, 2000 noisy archive images were randomly selected from the validation set for comparison between manual annotation and model classification results. Two strategies, random denoising (using median filtering without distinguishing noise types) and classification guided denoising (using this model method), were used for comparative experiments. The results are shown in Table 3. It can be seen that the classification guided denoising strategy improves the recall rate of text detection by 5.4% compared to the random denoising strategy, and the Top-1 accuracy and mAP increase by 3.2% and 3.1%, respectively. This indicates that accurate noise classification can adopt adaptive filtering methods for different types of noise, thereby more effectively restoring image quality and improving the performance of downstream text detection and retrieval tasks. In actual archival images, noise types often appear mixed. At this time, the system will prioritize salt and pepper noise detection and median filtering, and then perform Gaussian noise judgment and Gaussian filtering on the remaining noise to form a cascaded denoising process. This design ensures processing efficiency while enhancing denoising robustness in complex noisy scenes.

Table 3 Comparison of effectiveness results of noise classification methods

| Denoising strategy | Noise classification accuracy (%) | Text detection recall rate (%) | Top-1 accuracy (%) | mAP%) |
| --- | --- | --- | --- | --- |
| Random denoising | / | 86.3 | 80.5 | 85.2 |
| Classification guided denoising | 94.2 | 91.7 | 83.7 | 88.3 |

The use of simple feature concatenation in the GAT attention weight calculation is not justified against more sophisticated relational reasoning alternatives.

Reply: Thank you very much for your comments. The comparison between feature stitching and other alternative techniques has been supplemented as follows.

To verify the rationality of the feature concatenation method, this study compared it with two mainstream relational inference methods on the validation set, namely the Relationship Graph Convolutional Network (R-GCN) and the Transformer based Relationship Inference Module (RelTransformer). The results are shown in Table 4. It can be found that the feature stitching method has a difference of no more than 0.3% in Top-1 accuracy and mAP compared to R-GCN and RelTransformer, and the retrieval accuracy of the three methods is basically the same. However, in terms of retrieval efficiency, the single response time of the feature stitching method is 52.6ms, while R-GCN and RelTransformer increase to 71.2ms and 89.5ms, respectively, which are 35.4% and 70.2% higher than the feature stitching method. This efficiency difference will be significantly amplified in large-scale archive retrieval scenarios, directly affecting the real-time response capability of the system. In addition, the relationship types in the archive scene diagram have highly structured and finite characteristics. According to the annotation statistics of the dataset, the relationships between archive elements can be clearly classified into four types: inclusion relationships, location relationships, association relationships, and membership relationships. Each type of relationship has clear boundaries and low ambiguity in semantics. In such a highly standardized semantic space, feature concatenation and multi classification through fully connected layers are sufficient to fully capture the association patterns between node pairs. The introduction of more complex inference modules brings extremely limited accuracy gains, but at the cost of significant efficiency losses. Therefore, the study chose feature concatenation as the basic method for relationship prediction, which meets the efficient retrieval needs of large-scale archives while ensuring retrieval accuracy, achieving the best balance between accuracy and efficiency.

Table 4 Comparison of results from different feature splicing methods

| Relational reasoning method | Top-1 accuracy (%) | mAP (%) | Single search response time (ms) |
| --- | --- | --- | --- |
| Feature concatenation (in this study) | 83.5 | 88.1 | 52.6 |
| R-GCN | 83.7 | 88.3 | 71.2 |
| RelTransformer | 83.8 | 88.4 | 89.5 |

The transition from local node-level weighting to global scene graph feature pooling via average pooling is not justified compared to other pooling strategies.

Reply: Thank you very much for your comments. The reason for choosing average pooling as the aggregation method from node level features to global scene graph features has been added.

The number of nodes contained in different archive images varies greatly. For example, a simple archive may only contain 2-3 nodes, while a complex archive may contain more than 10 nodes, and the contribution of each node to the retrieval task is different. To convert these variable length node level feature sequences into fixed dimensional global scene graph features, pooling operations are required for aggregation. The study chose average pooling as the aggregation method, which focuses on calculating the mean of the weighted feature vectors of all nodes in the node dimension. This strategy can uniformly integrate the information of all nodes, avoiding the dominance of global representation due to significant local features of certain nodes, thereby preserving the overall structural semantics. In contrast, max pooling only retains the maximum values of node features in each dimension. Although it can highlight the most significant local responses, it may lose the distribution information of other nodes, especially when there are multiple equally important key nodes in the archive image. Max pooling may lead to information bias. In addition, average pooling is less sensitive to fluctuations in node features and can more stably reflect the overall structure of the scene graph in situations where the number of nodes varies greatly.

The cross-modal attention calculation does not address potential scale discrepancies between scene graph and text features before similarity computation.

Reply: Thank you very much for your comments. This study has implicitly addressed the issue of feature scale differences through various mechanisms. Firstly, when calculating the cross modal attention matrix, the study introduced the mean normalization operation of feature dimensions, which not only alleviates the problem of gradient vanishing caused by excessive inner product, but also balances the scale differences of different modal features to a certain extent. Then, the scene graph features are extracted through the GAT network, and the text features are encoded through the BERT model. Both paths contain layer normalization mechanisms. The attention calculation and graph convolution operations in GAT have scale adaptive capabilities, while the layer normalization in BERT can effectively stabilize the feature distribution, making the two types of features within a relatively standardized scale range before entering cross modal fusion. Finally, the cross modal fusion adopts a weighted sum method, and the attention weights are automatically learned based on the similarity matrix. This soft alignment mechanism can adaptively adjust the contribution of different modal features, thereby alleviating the impact caused by scale differences.

The choice of 100 cluster centers and 10 neighboring clusters for FAISS indexing lacks a sensitivity analysis or empirical justification.

Reply: Thank you for your feedback on the manuscript. Under your guidance, the sensitivity analysis has been supplemented as follows.

To determine the optimal configuration of the number of cluster centers and query neighborhoods in the FAISS vector index, sensitivity analysis was conducted on the validation set. The number of cluster centers is set to 50, 100, and 150, respectively, and the number of query neighborhoods is set to 5, 10, and 20, respectively. The results are shown in Table 1. It can be observed that when the number of cluster centers is 50, the discriminative power of feature distribution is insufficient. Although the response time is short, the Top-1 accuracy is relatively low; When the number of cluster centers increases to 100, the accuracy significantly improves while the response time remains within an acceptable range; When it continues to increase to 150, the accuracy improvement tends to saturate, while the response time increases significantly. In terms of the number of query neighborhoods, when the number of query neighborhoods increases from 5 to 10, the accuracy steadily improves, but the increase in response time is limited; When further increased to 20, the accuracy improvement was weak, but the response time was significantly prolonged. Taking into account both retrieval accuracy and efficiency, this study selected a parameter configuration of 100 cluster centers and 10 query neighborhoods as the FAISS index. This configuration maintains high retrieval accuracy while controlling the response time of a single retrieval to 52.6ms, meeting the real-time retrieval needs of large-scale archives. The results of parameter sensitivity analysis have been shown in Table 1.

Table 1 Parameter sensitivity analysis

| Number of Cluster Centers | Query the number of neighborhoods | Top-1 accuracy (%) | Single search response time (ms) |
| --- | --- | --- | --- |
| 50 | 5 | 81.6 | 46.2 |
|  | 10 | 82.4 | 49.5 |
|  | 20 | 82.7 | 55.8 |
| 100 | 5 | 83.1 | 50.3 |
|  | 10 | 83.5 | 52.6 |
|  | 20 | 83.6 | 59.4 |
| 150 | 5 | 83.2 | 54.7 |
|  | 10 | 83.5 | 58.2 |
|  | 20 | 83.7 | 65.1 |

The cluster number configuration was determined based on pre-experiments on the validation set: if the number of cluster centers was too small (such as 50), it would lead to insufficient discrimination in feature distribution and reduce the retrieval recall rate. If there was too much (such as 150), redundant computation would be introduced, increasing the retrieval delay. Experiments showed that, under the current dataset scale and feature distribution, when the number of cluster centers was 100 and the number of query clusters was 10, it was possible to maintain a high retrieval accuracy while stably controlling the response time of a single retrieval within the target range, achieving the best trade-off between retrieval efficiency and effect.

The multi-task loss weights (λ1, λ2, λ3) are not specified, nor is the tuning process described.

Reply: Thank you very much for your comments. The process of setting and adjusting multi task loss weights has been supplemented as follows:

The loss weights for scene graph generation, cross modal fusion, and retrieval matching were set to 0.5, 0.3, and 0.2, respectively. This weight combination was determined through grid search optimization on the validation set: the search range was from 0.1 to 0.7 for each weight, with a step size of 0.1, for a total of 343 weight combinations. The experimental results showed that when the loss weight of scene graph generation was too high (such as 0.7 or above), the model focused too much on element detection and relationship prediction tasks, resulting in insufficient cross modal feature alignment ability, and the average accuracy on the validation set decreased by about 2.5%; When the weight of the retrieval matching loss was too high (such as 0.4 or above), the model was prone to overfitting. Although the retrieval matching loss decreased rapidly, the generalization ability weakened, and the Top-1 accuracy on the validation set fluctuated significantly. By comprehensively comparing the average accuracy of each weight combination with the Top-1 accuracy, 0.5:0.3:0.2 was determined as the optimal weight configuration. At this point, the average accuracy of the model on the validation set reached 88.1%, the Top-1 accuracy reached 83.5%, and the training process converged stably. This weight setting ensured the dominant position of the scene graph generation module as the basic feature extraction task, while also taking into account the optimization requirements of cross modal feature alignment and retrieval matching tasks, achieving an effective balance of multi task learning objectives.

The dataset's annotation density and the criteria for "similarity labels" (levels 1-5) are not clearly defined.

Reply: Thank you very much for your comments. The dataset's annotation density and the criteria for "similarity labels" (levels 1-5) have been clearly defined as follows:

All images were annotated at multiple levels. At the node level, four core elements including text blocks, seals, tables, and blank areas were annotated, totaling 182000 nodes. On average, each image contains about 3.6 nodes, with text block nodes accounting for the highest proportion, about 62%. At the relational level, four types of semantic relationships were annotated, including relationships, orientation relationships, association relationships, and membership relationships, totaling 256000 relationships. On average, each image contained about 5.1 relationships, with orientation relationships and association relationships being the most common, accounting for 38% and 29% of the total correlation coefficients, respectively. The above annotation density ensured the integrity and semantic richness of scene graph construction. The retrieval similarity tags adopted a five level scoring system, and were independently annotated by three experts in the field of archives based on the correlation between query conditions and archive images. The final tags were based on the consensus of the majority or expert consultation results. The specific definitions of each level were as follows: Level 1 (completely unrelated) indicated that there was no correlation between the image content and the query conditions in terms of theme, elements, and structure, such as when querying the "2020 Financial Audit Report" and returning the "1985 Personnel Appointment and Removal Notice"; Level 2 (partially related) indicated that the image only had a very small number of common elements with the query criteria, but the overall theme and structure did not match, such as the query containing "seals" but returning images with only similar seals and no matching text; Level 3 (moderately correlated) indicated that the image matched the query criteria on some key elements, but there were significant differences in overall structure or semantic associations, such as matching table formats but not matching document numbers; Level 4 (highly correlated) indicated a high degree of match between the image and the query criteria in terms of primary elements and structure, with only minor differences in details such as title matching but slight offset in seal position; Level 5 (completely related) indicated that the image and query conditions were completely consistent in terms of text content, visual elements, layout structure, and semantic relationships. This grading standard provided a clear benchmark for evaluating retrieval performance, ensuring the consistency and reliability of evaluation results.

The ablation study results are presented without error bars or confidence intervals to demonstrate statistical significance.

Reply: Thank you very much for your comments. The statistical testing of the ablation experiment has been supplemented, as follows:

Table 2 shows the results of ablation experiments using scene graph structure matching optimization. The study repeated the experiment for 5 times to assess each indicator. After introducing scene graph structure matching optimization, the retrieval accuracy of the model was significantly improved. The Top-1 accuracy increased from 80.1% ± 0.42% to 83.7% ± 0.38%, and the average accuracy increased from 85.6% ± 0.45% to 88.3% ± 0.41%. Through paired t-test verification, the above indicators showed statistically significant improvements (p<0.01). Structural matching optimization effectively distinguished archive images with similar features but different structures by fusing logical information of nodes and relationships, thereby improving retrieval accuracy. Although the retrieval response time increased by 4.3ms, it still remained within 60ms, meeting the real-time requirements of archive retrieval. This result indicated that scene graph structure matching optimization significantly improved the retrieval performance of the model at an acceptable response time cost, verifying its effectiveness and necessity in fine-grained retrieval.

Table 2 Ablation experiment results of scene graph structure matching optimization

| Index | Third level attention fusion+unstructured matching | Three level attention fusion+scene graph structure matching |
| --- | --- | --- |
| Top-1 accuracy (%) | 80.1±0.42 | 83.7±0.38 |
| Top-5 accuracy (%) | 92.5±0.31 | 94.2±0.29 |
| Top-10 accuracy (%) | 96.2±0.24 | 97.5±0.22 |
| mAP（%） | 85.6±0.455 | 88.3±0.41 |
| F1 value (%) | 82.9±0.51 | 85.9±0.47 |
| Search response time (ms) | 48.3±1.24 | 52.6±1.31 |

The comparison against OCR+TF-IDF shows a large margin, but this is a weak baseline; the improvement over the more competitive ARNet is modest.

Reply: Thank you very much for your comments. Compared with ARNet, the advantages of this study have been supplemented as follows.

Thirdly, compared with advanced models such as CLIP and ARNet, the proposed model maintained an accuracy advantage of 3% -7%. Specifically, while ARNet performs effectively in general archive retrieval tasks, it does not explicitly model the logical relationships between elements, which limits its ability to distinguish archives that have different structures yet remain visually similar. In contrast, the proposed model utilizes these structured semantics to improve fine-grained retrieval performance, especially in scenarios involving sealed occlusion and small text blocks, with an improvement of over 6%.

The fine-grained retrieval case studies rely on qualitative interpretation without explaining what the feature similarity scores actually encode.

Reply: Thank you very much for your comments. The feature similarity scores actually encode has been explained as follows:

To further verify the fine-grained retrieval capability of the model, the experiment selected three typical archive retrieval scenarios for case analysis, namely: (1) official seal obscured text retrieval (querying for title text with official seal obscuration); (2) small text block retrieval (querying for archives with numbered small text blocks); and (3) multi-element association retrieval (querying for archives with specific tables + subordinate numbers). The feature similarity reflects the degree of matching between the query and the candidate archive in both visual and textual dimensions, with higher values indicating closer proximity in key elements, semantic content, and cross modal associations. The structural similarity is calculated based on the consistency of nodes and relationships in the scene graph, as shown in formula (11), reflecting the degree of matching between the image layout structure and semantic logic.

The results of the official seal obscured text retrieval case are shown in Table 6. The query conditions were that the title contained the 2020 annual financial audit report and the official seal obscured the right 1/3 area of the title. The results showed that in the scenario where the official seal obscured the title, the proposed model could accurately identify the target file (A00125) and rank it first, with a final score of 91.5%, which was higher than other candidate files. Although A00512 had a high text feature similarity, its structural similarity was low due to the lack of official seal obscuration, and it was finally ranked third. A00943 had a high structural similarity, but a low text feature similarity, and was finally ranked fifth. This showed that the model could effectively distinguish files with similar texts but different occlusion structures through the weighted fusion of feature similarity and structural similarity, verifying the model's fine-grained retrieval capability for official seal occlusion scenarios.

The dataset limitation to a single provincial archive is noted, but potential annotation biases and category representativeness are not addressed.

Reply: Thank you very much for your comments. The potential annotation biases and category representativeness have been supplemented as follows:

All images were annotated at multiple levels. At the node level, four core elements including text blocks, seals, tables, and blank areas were annotated, totaling 182000 nodes. On average, each image contains about 3.6 nodes, with text block nodes accounting for the highest proportion, about 62%. At the relational level, four types of semantic relationships were annotated, including relationships, orientation relationships, association relationships, and membership relationships, totaling 256000 relationships. On average, each image contained about 5.1 relationships, with orientation relationships and association relationships being the most common, accounting for 38% and 29% of the total correlation coefficients, respectively. The above annotation density ensured the integrity and semantic richness of scene graph construction. The retrieval similarity tags adopted a five level scoring system, and were independently annotated by three experts in the field of archives based on the correlation between query conditions and archive images. The final tags were based on the consensus of the majority or expert consultation results. The specific definitions of each level were as follows: Level 1 (completely unrelated) indicated that there was no correlation between the image content and the query conditions in terms of theme, elements, and structure, such as when querying the "2020 Financial Audit Report" and returning the "1985 Personnel Appointment and Removal Notice"; Level 2 (partially related) indicated that the image only had a very small number of common elements with the query criteria, but the overall theme and structure did not match, such as the query containing "seals" but returning images with only similar seals and no matching text; Level 3 (moderately correlated) indicated that the image matched the query criteria on some key elements, but there were significant differences in overall structure or semantic associations, such as matching table formats but not matching document numbers; Level 4 (highly correlated) indicated a high degree of match between the image and the query criteria in terms of primary elements and structure, with only minor differences in details such as title matching but slight offset in seal position; Level 5 (completely related) indicated that the image and query conditions were completely consistent in terms of text content, visual elements, layout structure, and semantic relationships. This grading standard provided a clear benchmark for evaluating retrieval performance, ensuring the consistency and reliability of evaluation results.

This study has two limitations: First, the dataset came from a single provincial archive. While the archive types covered were representative, they did not cover special scenarios such as historical handwritten archives and multilingual archives, requiring further verification of the model's universality. Second, while the index construction time met the requirements of a million-level archive, it is still longer than that of OCR+TF-IDF, and the indexing efficiency of large-scale archives needs to be optimized. Future research could be advanced in two ways: first, expanding the dataset to include handwritten and multilingual archives, optimizing the texture features of scene graph nodes and the text recognition module; and second, introducing quantitative indexing technology to shorten index construction time while ensuring accuracy.

The conclusion overstates practical readiness without reiterating the limitations regarding dataset diversity and indexing time.

Reply: Thank you very much for your comments. The conclusion section has been revised and the limitations regarding dataset diversity and indexing time have been reiterated. Please see the revised conclusion section as follows:

5. Conclusion

Aiming at the demand for visual-text dual feature fusion of archival text images, the study constructed a five-order fine-grained retrieval model consisting of input layer, preprocessing layer, scene graph generation layer, attention fusion layer, and retrieval matching layer. The archive-specific scene graph generation module effectively improved the ability to capture domain features. The experimental results showed that the model achieved a Top-1 accuracy of 83.7% and an mAP of 88.3% on the test set, demonstrating excellent fine-grained retrieval performance in typical scenarios such as seal occlusion, small text blocks, and multi-element association. The archive specific scene graph generation module effectively improved the ability to capture domain features. Through customized node definitions, prior box optimization, and structural simplification, the accuracy increased by 12.2% compared to the Top-1 of general scene graphs, solving the problem of detecting small text blocks and seal occlusions. Although the model achieved good performance in retrieval accuracy and response speed, there are still certain limitations. Although the average indexing time of the model meets the requirements of building millions of archives, the indexing efficiency in large-scale dynamic update scenarios needs to be optimized. Further optimization can be carried out in future research. In terms of handwritten archives, a multi lingual pre trained model based on convolutional recurrent networks and linked temporal classification can be introduced as the text encoding basis. Language identification attributes can be introduced in scene graph nodes to construct a cross lingual semantic alignment module. Finally, the inverted index compression technique based on product quantization can shorten the index construction time while ensuring retrieval accuracy.

The proposed future work on handwritten and multilingual archives is vague and lacks specific technical challenges to guide subsequent research.

Reply: Thank you very much for your comments. The discussion on further research in the future has been revised as follows.

Further optimization can be carried out in future research. In terms of handwritten archives, a multi lingual pre trained model based on convolutional recurrent networks and linked temporal classification can be introduced as the text encoding basis. Language identification attributes can be introduced in scene graph nodes to construct a cross lingual semantic alignment module. Finally, the inverted index compression technique based on product quantization can shorten the index construction time while ensuring retrieval accuracy.
