# Supplementary material for: Construction of a fine-grained retrieval model for archival text-image based on the integration of scene graph generation and attention mechanism
Source: PLoS One. 2026 Jul 24;21(7):e0353505. doi: 10.1371/journal.pone.0353505 (PMC13399324; doi:10.1371/journal.pone.0353505)
Supplement: Peer review history

# Peer review: Decision Letter 0

**DOI:** 10.1371/journal.pone.0353505.r001

**Authors:** Rehan Ashraf

16 Feb 2026

PONE-D-25-67761

Construction of a Fine-Grained Retrieval Model for Archival Text-Image Based on the Integration of Scene Graph Generation and Attention Mechanism

PLOS One

Dear Dr. Zhang,

Thank you for submitting your manuscript to PLOS ONE. After careful consideration, we feel that it has merit but does not fully meet PLOS ONE’s publication criteria as it currently stands. Therefore, we invite you to submit a revised version of the manuscript that addresses the points raised during the review process.

Please submit your revised manuscript by Apr 02 2026 11:59PM. If you will need more time than this to complete your revisions, please reply to this message or contact the journal office at plosone@plos.org. When you're ready to submit your revision, log on to https://www.editorialmanager.com/pone/ and select the 'Submissions Needing Revision' folder to locate your manuscript file.

Please include the following items when submitting your revised manuscript:

- A letter that responds to each point raised by the academic editor and reviewer(s). You should upload this letter as a separate file labeled 'Response to Reviewers'.
- A marked-up copy of your manuscript that highlights changes made to the original version. You should upload this as a separate file labeled 'Revised Manuscript with Track Changes'.
- An unmarked version of your revised paper without tracked changes. You should upload this as a separate file labeled 'Manuscript

If you would like to make changes to your financial disclosure, please include your updated statement in your cover letter. Guidelines for resubmitting your figure files are available below the reviewer comments at the end of this letter.

If applicable, we recommend that you deposit your laboratory protocols in protocols.io to enhance the reproducibility of your results. Protocols.io assigns your protocol its own identifier (DOI) so that it can be cited independently in the future. For instructions see: https://journals.plos.org/plosone/s/submission-guidelines#loc-laboratory-protocols. Additionally, PLOS ONE offers an option for publishing peer-reviewed Lab Protocol articles, which describe protocols hosted on protocols.io. Read more information on sharing protocols at https://plos.org/protocols?utm_medium=editorial-email&utm_source=authorletters&utm_campaign=protocols.

We look forward to receiving your revised manuscript.

Kind regards,

Rehan Ashraf, Ph.D

Academic Editor

PLOS One

Journal Requirements:

When submitting your revision, we need you to address these additional requirements.

1. Please ensure that your manuscript meets PLOS ONE's style requirements, including those for file naming. The PLOS ONE style templates can be found at

https://journals.plos.org/plosone/s/file?id=wjVg/PLOSOne_formatting_sample_main_body.pdf and

https://journals.plos.org/plosone/s/file?id=ba62/PLOSOne_formatting_sample_title_authors_affiliations.pdf

2. Please note that PLOS One has specific guidelines on code sharing for submissions in which author-generated code underpins the findings in the manuscript. In these cases, we expect all author-generated code to be made available without restrictions upon publication of the work. Please review our guidelines at https://journals.plos.org/plosone/s/materials-and-software-sharing#loc-sharing-code and ensure that your code is shared in a way that follows best practice and facilitates reproducibility and reuse.

3. Please note that PLOS One has specific guidelines on code sharing for submissions in which author-generated code underpins the findings in the manuscript. In these cases, we expect all author-generated code to be made available without restrictions upon publication of the work. Please review our guidelines at https://journals.plos.org/plosone/s/materials-and-software-sharing#loc-sharing-code and ensure that your code is shared in a way that follows best practice and facilitates reproducibility and reuse.

4. We note that your Data Availability Statement is currently as follows: “All relevant data are within the manuscript and its Supporting Information files.”

Please confirm at this time whether or not your submission contains all raw data required to replicate the results of your study. Authors must share the “minimal data set” for their submission. PLOS defines the minimal data set to consist of the data required to replicate all study findings reported in the article, as well as related metadata and methods (https://journals.plos.org/plosone/s/data-availability#loc-minimal-data-set-definition).

For example, authors should submit the following data:

- The values behind the means, standard deviations and other measures reported;

- The values used to build graphs;

- The points extracted from images for analysis.

Authors do not need to submit their entire data set if only a portion of the data was used in the reported study.

If your submission does not contain these data, please either upload them as Supporting Information files or deposit them to a stable, public repository and provide us with the relevant URLs, DOIs, or accession numbers. For a list of recommended repositories, please see https://journals.plos.org/plosone/s/recommended-repositories.

If there are ethical or legal restrictions on sharing a de-identified data set, please explain them in detail (e.g., data contain potentially sensitive information, data are owned by a third-party organization, etc.) and who has imposed them (e.g., an ethics committee). Please also provide contact information for a data access committee, ethics committee, or other institutional body to which data requests may be sent. If data are owned by a third party, please indicate how others may request data access.

5. If the reviewer comments include a recommendation to cite specific previously published works, please review and evaluate these publications to determine whether they are relevant and should be cited. There is no requirement to cite these works unless the editor has indicated otherwise.

[Note: HTML markup is below. Please do not edit.]

Reviewers' comments:

Reviewer's Responses to Questions

**Comments to the Author**

1. Is the manuscript technically sound, and do the data support the conclusions?

The manuscript must describe a technically sound piece of scientific research with data that supports the conclusions. Experiments must have been conducted rigorously, with appropriate controls, replication, and sample sizes. The conclusions must be drawn appropriately based on the data presented.

Reviewer #1: Yes

Reviewer #2: Partly

**********

2. Has the statistical analysis been performed appropriately and rigorously?

Reviewer #1: Yes

Reviewer #2: No

**********

3. Have the authors made all data underlying the findings in their manuscript fully available?

The PLOS Data policy requires authors to make all data underlying the findings described in their manuscript fully available without restriction, with rare exception (please refer to the Data Availability Statement in the manuscript PDF file). The data should be provided as part of the manuscript or its supporting information, or deposited to a public repository. For example, in addition to summary statistics, the data points behind means, medians and variance measures should be available. If there are restrictions on publicly sharing data—e.g. participant privacy or use of data from a third party—those must be specified.

Reviewer #1: No

Reviewer #2: Yes

**********

4. Is the manuscript presented in an intelligible fashion and written in standard English?

PLOS ONE does not copyedit accepted manuscripts, so the language in submitted articles must be clear, correct, and unambiguous. Any typographical or grammatical errors should be corrected at revision, so please note any specific errors here.

Reviewer #1: Yes

Reviewer #2: No

**********

5. Review Comments to the Author

Please use the space provided to explain your answers to the questions above. You may also include additional comments for the author, including concerns about dual publication, research ethics, or publication ethics. (Please upload your review as an attachment if it exceeds 20,000 characters)

Reviewer #1: This study proposes a fine-grained retrieval model for archival text-images by integrating archive-specific scene graph generation with a three-level attention fusion mechanism. The model consists of five layers: input, preprocessing, scene graph generation, attention fusion, and retrieval matching.

The core innovation lies in:

Designing an archive-specific scene graph module to better detect and represent key archival elements (e.g., seals, small text blocks, tables, serial numbers).

Introducing a three-level attention mechanism (scene graph attention, text attention using BERT, and cross-modal attention) to align visual and textual features.

Combining feature similarity and structural graph matching to improve retrieval ranking.

Experiments conducted on a dataset of 50,000 archival images demonstrate that the proposed model achieves 83.7% Top-1 accuracy and 88.3% mAP, outperforming OCR+TF-IDF, SceneGraph+CNN, CLIP, and ARNet models. The model also shows robustness in complex scenarios such as official seal occlusion and small text blocks, while maintaining acceptable retrieval efficiency.

Strengths

+Clear research motivation: The paper clearly addresses the practical challenges of archival retrieval, especially noise, seal occlusion, and small text blocks.

+Well-structured methodology: The five-layer architecture is logically organized and systematically described.

+Domain-specific innovation: The archive-specific scene graph (custom node definitions and prior box optimization) is a meaningful domain adaptation.

+Comprehensive technical design: Includes detailed formulations for graph construction, attention mechanisms, similarity computation, and multi-task loss.

+Strong experimental validation:

++Large real-world dataset (50,000 images).

++Multiple evaluation metrics (Top-1/5/10, mAP, F1, response time).

++Ablation studies validating each core module.

++Comparisons with strong baselines (CLIP, ARNet).

+Case study analysis: Detailed fine-grained retrieval cases (seal occlusion, small text block, multi-element association) enhance practical relevance.

+Balanced accuracy and efficiency analysis: Includes index construction time and large-scale retrieval response time.

+Discussion and limitations provided: The authors explicitly acknowledge dataset and indexing limitations.

Weaknesses

-Writing clarity and language quality: Some grammatical inconsistencies and awkward phrasing reduce readability. The manuscript would benefit from professional language editing.

-Overly dense methodological section: The large number of equations and technical details may obscure the main conceptual contributions.

-Limited dataset diversity: Data originate from a single provincial archive, which may limit generalizability.

-Recent references dominate: Many citations are from 2024–2025, and foundational works in scene graph generation and cross-modal retrieval could be cited more comprehensively.

-Hyperparameter justification is limited: Some parameter choices (e.g., weighting 0.7/0.3 in final score fusion) are not deeply analyzed.

-Figure presentation: Some figures are crowded and could be improved for clarity and visual consistency.

-Reproducibility details: Although dataset statistics are provided, public availability of data/code is not clearly specified in the manuscript.

Overall Recommendation

-The manuscript presents a technically solid and experimentally validated contribution to fine-grained archival retrieval. The integration of archive-specific scene graph modeling with multi-level attention fusion is meaningful and well-supported by experiments.

The identified weaknesses are primarily related to clarity, presentation, and minor methodological justification, rather than fundamental flaws in design or validation.

Recommendation: Minor revisions.

Suggested revisions:

Improve language quality and readability.

Clarify parameter selection rationale (e.g., fusion weights).

Strengthen discussion on generalizability and dataset diversity.

Improve figure clarity and formatting.

Clarify data/code availability for reproducibility.

With these refinements, the paper would be suitable for publication.

Reviewer #2: The introduction cites recent papers but does not critically engage with their limitations to clearly establish the unique contribution of this study.

The description of "prior box" optimization lacks empirical data or analysis from the dataset to justify the chosen scales and aspect ratios.

The noise classification method in the preprocessing layer is mentioned but the specific statistical model and its accuracy are not explained.

The use of simple feature concatenation in the GAT attention weight calculation is not justified against more sophisticated relational reasoning alternatives.

The transition from local node-level weighting to global scene graph feature pooling via average pooling is not justified compared to other pooling strategies.

The cross-modal attention calculation does not address potential scale discrepancies between scene graph and text features before similarity computation.

The choice of 100 cluster centers and 10 neighboring clusters for FAISS indexing lacks a sensitivity analysis or empirical justification.

The multi-task loss weights (λ1, λ2, λ3) are not specified, nor is the tuning process described.

The dataset's annotation density and the criteria for "similarity labels" (levels 1-5) are not clearly defined.

The ablation study results are presented without error bars or confidence intervals to demonstrate statistical significance.

The comparison against OCR+TF-IDF shows a large margin, but this is a weak baseline; the improvement over the more competitive ARNet is modest.

The fine-grained retrieval case studies rely on qualitative interpretation without explaining what the feature similarity scores actually encode.

The dataset limitation to a single provincial archive is noted, but potential annotation biases and category representativeness are not addressed.

The conclusion overstates practical readiness without reiterating the limitations regarding dataset diversity and indexing time.

The proposed future work on handwritten and multilingual archives is vague and lacks specific technical challenges to guide subsequent research.

**********

6. PLOS authors have the option to publish the peer review history of their article (what does this mean?). If published, this will include your full peer review and any attached files.

If you choose “no”, your identity will remain anonymous but your review may still be made public.

**Do you want your identity to be public for this peer review?**  For information about this choice, including consent withdrawal, please see our Privacy Policy.

Reviewer #1: No

Reviewer #2: No

**********

[NOTE: If reviewer comments were submitted as an attachment file, they will be attached to this email and accessible via the submission site. Please log into your account, locate the manuscript record, and check for the action link "View Attachments". If this link does not appear, there are no attachment files.]

To ensure your figures meet our technical requirements, please review our figure guidelines: https://journals.plos.org/plosone/s/figures

You may also use PLOS’s free figure tool, NAAS, to help you prepare publication quality figures: https://journals.plos.org/plosone/s/figures#loc-tools-for-figure-preparation.

NAAS will assess whether your figures meet our technical requirements by comparing each figure against our figure specifications.

# Author comment: Author response to Decision Letter 1

**DOI:** 10.1371/journal.pone.0353505.r002

7 May 2026

The manuscript has been revised according to comments

Attachment Submitted filename: Response to comments.doc

# Peer review: Decision Letter 1

**DOI:** 10.1371/journal.pone.0353505.r003

**Authors:** Rehan Ashraf

24 Jun 2026

Construction of a Fine-Grained Retrieval Model for Archival Text-Image Based on the Integration of Scene Graph Generation and Attention Mechanism

PONE-D-25-67761R1

Dear Dr. Zhang,

We’re pleased to inform you that your manuscript has been judged scientifically suitable for publication and will be formally accepted for publication once it meets all outstanding technical requirements.

Within one week, you’ll receive an e-mail detailing the required amendments. When these have been addressed, you’ll receive a formal acceptance letter and your manuscript will be scheduled for publication.

An invoice will be generated when your article is formally accepted. Please note, if your institution has a publishing partnership with PLOS and your article meets the relevant criteria, all or part of your publication costs will be covered. Please make sure your user information is up-to-date by logging into Editorial Manager at Editorial Manager® and clicking the ‘Update My Information' link at the top of the page. For questions related to billing, please contact billing support.

If your institution or institutions have a press office, please notify them about your upcoming paper to help maximize its impact. If they’ll be preparing press materials, please inform our press team as soon as possible -- no later than 48 hours after receiving the formal acceptance. Your manuscript will remain under strict press embargo until 2 pm Eastern Time on the date of publication. For more information, please contact onepress@plos.org.

Kind regards,

Rehan Ashraf, Ph.D

Academic Editor

PLOS One

Additional Editor Comments (optional):

Reviewers' comments:

Reviewer's Responses to Questions

**Comments to the Author**

1. If the authors have adequately addressed your comments raised in a previous round of review and you feel that this manuscript is now acceptable for publication, you may indicate that here to bypass the “Comments to the Author” section, enter your conflict of interest statement in the “Confidential to Editor” section, and submit your "Accept" recommendation.

Reviewer #3: (No Response)

Reviewer #4: (No Response)

**********

2. Is the manuscript technically sound, and do the data support the conclusions?

The manuscript must describe a technically sound piece of scientific research with data that supports the conclusions. Experiments must have been conducted rigorously, with appropriate controls, replication, and sample sizes. The conclusions must be drawn appropriately based on the data presented.

Reviewer #3: (No Response)

Reviewer #4: (No Response)

**********

3. Has the statistical analysis been performed appropriately and rigorously?

Reviewer #3: (No Response)

Reviewer #4: (No Response)

**********

4. Have the authors made all data underlying the findings in their manuscript fully available?

The PLOS Data policy requires authors to make all data underlying the findings described in their manuscript fully available without restriction, with rare exception (please refer to the Data Availability Statement in the manuscript PDF file). The data should be provided as part of the manuscript or its supporting information, or deposited to a public repository. For example, in addition to summary statistics, the data points behind means, medians and variance measures should be available. If there are restrictions on publicly sharing data—e.g. participant privacy or use of data from a third party—those must be specified.

Reviewer #3: (No Response)

Reviewer #4: (No Response)

**********

5. Is the manuscript presented in an intelligible fashion and written in standard English?

PLOS ONE does not copyedit accepted manuscripts, so the language in submitted articles must be clear, correct, and unambiguous. Any typographical or grammatical errors should be corrected at revision, so please note any specific errors here.

Reviewer #3: (No Response)

Reviewer #4: (No Response)

**********

6. Review Comments to the Author

Please use the space provided to explain your answers to the questions above. You may also include additional comments for the author, including concerns about dual publication, research ethics, or publication ethics. (Please upload your review as an attachment if it exceeds 20,000 characters)

Reviewer #3: (No Response)

Reviewer #4: (No Response)

**********

7. PLOS authors have the option to publish the peer review history of their article (what does this mean?). If published, this will include your full peer review and any attached files.

If you choose “no”, your identity will remain anonymous but your review may still be made public.

**Do you want your identity to be public for this peer review?**  For information about this choice, including consent withdrawal, please see our Privacy Policy.

Reviewer #3: No

Reviewer #4: No

**********

# Editor report: Acceptance letter

**DOI:** 10.1371/journal.pone.0353505.r004

**Authors:** Rehan Ashraf

PONE-D-25-67761R1

PLOS One

Dear Dr. Zhang,

I'm pleased to inform you that your manuscript has been deemed suitable for publication in PLOS One. Congratulations! Your manuscript is now being handed over to our production team.

At this stage, our production department will prepare your paper for publication. This includes ensuring the following:

* All references, tables, and figures are properly cited

* All relevant supporting information is included in the manuscript submission,

* There are no issues that prevent the paper from being properly typeset

You will receive further instructions from the production team, including instructions on how to review your proof when it is ready. Please keep in mind that we are working through a large volume of accepted articles, so please give us a few days to review your paper and let you know the next and final steps.

Lastly, if your institution or institutions have a press office, please let them know about your upcoming paper now to help maximize its impact. If they'll be preparing press materials, please inform our press team within the next 48 hours. Your manuscript will remain under strict press embargo until 2 pm Eastern Time on the date of publication. For more information, please contact onepress@plos.org.

You will receive an invoice from PLOS for your publication fee after your manuscript has reached the completed accept phase. If you receive an email requesting payment before acceptance or for any other service, this may be a phishing scheme. Learn how to identify phishing emails and protect your accounts at https://explore.plos.org/phishing.

If we can help with anything else, please email us at customercare@plos.org.

Thank you for submitting your work to PLOS ONE and supporting open access.

Kind regards,

PLOS ONE Editorial Office Staff

on behalf of

Dr. Rehan Ashraf

Academic Editor

PLOS One
